# Higher odds of periodontitis in systemic lupus erythematosus compared to controls and rheumatoid arthritis: a systematic review, meta-analysis and network meta-analysis

**DOI:** 10.3389/fimmu.2024.1356714

**Published:** 2024-04-02

**Authors:** Ping Ren Tan, Aaron J. L. Lee, Joseph J. Zhao, Yiong Huak Chan, Jia Hui Fu, Margaret Ma, Sen Hee Tay

**Affiliations:** ^1^ Yong Loo Lin School of Medicine, National University of Singapore, Singapore, Singapore; ^2^ Lee Kong Chian School of Medicine, Nanyang Technological University, Singapore, Singapore; ^3^ Biostatistics Unit, Yong Loo Lin School of Medicine, National University of Singapore, Singapore, Singapore; ^4^ Faculty of Dentistry, National University of Singapore, Singapore, Singapore; ^5^ Division of Rheumatology, Department of Medicine, National University Hospital, Singapore, Singapore; ^6^ Department of Medicine, Yong Loo Lin School of Medicine, National University of Singapore, Singapore, Singapore

**Keywords:** periodontitis, rheumatoid arthritis, systemic lupus erythematosus, meta-analysis, network meta-analysis

## Abstract

**Introduction:**

Periodontitis as a comorbidity in systemic lupus erythematosus (SLE) is still not well recognized in the dental and rheumatology communities. A meta-analysis and network meta-analysis were thus performed to compare the (i) prevalence of periodontitis in SLE patients compared to those with rheumatoid arthritis (RA) and (ii) odds of developing periodontitis in controls, RA, and SLE.

**Methods:**

Pooled prevalence of and odds ratio (OR) for periodontitis were compared using meta-analysis and network meta-analysis (NMA).

**Results:**

Forty-three observational studies involving 7,800 SLE patients, 49,388 RA patients, and 766,323 controls were included in this meta-analysis. The pooled prevalence of periodontitis in SLE patients (67.0%, 95% confidence interval [CI] 57.0-77.0%) was comparable to that of RA (65%, 95% CI 55.0-75.0%) (p>0.05). Compared to controls, patients with SLE (OR=2.64, 95% CI 1.24-5.62, p<0.01) and RA (OR=1.81, 95% CI 1.25-2.64, p<0.01) were more likely to have periodontitis. Indirect comparisons through the NMA demonstrated that the odds of having periodontitis in SLE was 1.49 times higher compared to RA (OR=1.49, 95% CI 1.09-2.05, p<0.05).

**Discussion:**

Given that RA is the autoimmune disease classically associated with periodontal disease, the higher odds of having periodontitis in SLE are striking. These results highlight the importance of addressing the dental health needs of patients with SLE.

**Systematic review registration:**

https://www.crd.york.ac.uk/PROSPERO/ identifier CRD42021272876.

## 1 Introduction

Periodontitis is a microbially-associated, host-mediated hyperinflammatory condition that leads to the destruction of structures supporting the teeth, including the alveolar bone, periodontal ligament, and cementum (1–3). If not addressed, periodontitis can lead to early tooth loss, affecting one’s ability to chew, self-confidence, and overall well-being (4–6). Furthermore, periodontitis has been linked to broader health implications, contributing to conditions such as cardiovascular disease, type 2 diabetes mellitus, and adverse outcomes in pregnancy (7). The global prevalence of periodontitis is 20-50%, and together with gingivitis, its precursor, they collectively constitute the 11^th^ most prevalent condition worldwide (8). Its impact is substantial, accounting for 3.5 million years of disability and causing an estimated productivity loss of around USD$54 billion annually (4). Recent studies have also shown significant associations between rheumatoid arthritis (RA) and, to a smaller degree, systemic lupus erythematosus (SLE), with periodontitis (9–11). However, these associations remain underappreciated and warrant further investigations (12).

Rheumatoid arthritis is a chronic autoimmune disease that has both joint-specific and systemic manifestations (13). Its global prevalence, as estimated by the Global Burden of Disease 2010 Study, stands at approximately 0.24% (14, 15). SLE is a potentially fatal, chronic autoimmune disease that affects multiple systems. It primarily affects women, with the highest incidence during childbearing years (16). The global prevalence of SLE has been on the rise over the years, escalating from 40 cases per 100,000 individuals in the 1970s to 100 cases per 100,000 individuals since the 2000s (17).

The exact aetiopathogenesis of RA and SLE is complex, with multiple genetic, epigenetic, immunological, and environmental factors involved. These factors often culminate in immune dysregulation and autoantibody production (18–20). Moreover, evidence suggests that infections may contribute to the development of these diseases through mechanisms such as molecular mimicry and uncontrolled immune cell activation (21). The relationship between RA and periodontitis is better established than that between SLE and periodontitis, partly due to the process of citrullination. Citrullination involves the modification of arginine residues to citrulline by peptidyl arginine deiminases. *Porphyromonas gingivalis*, a key organism in periodontitis, is the sole prokaryotic organism that secretes *Porphyromonas gingivalis*-derived peptidyl arginine deiminase (PPAD) (11, 22). Prolonged exposure to citrullinated proteins in the oral cavity may trigger the production of anti-cyclic citrullinated peptide (CCP) antibodies, potentially leading to the onset of RA in susceptible individuals (23). The associations between (i) periodontitis and anti-CCP seropositivity and (ii) periodontitis severity and presence of anti-CCP antibodies in patients with RA provide further support for this hypothesis (24, 25). In addition, disease-modifying antirheumatic drugs ameliorate both RA and periodontitis, suggesting shared inflammatory pathways (26). In contrast, while a connection between SLE and periodontitis is emerging, it is still in the early stages of recognition, and the relationship has not been confirmed (10, 27, 28). Despite the high prevalence of *Porphyromonas gingivalis*, this pathogen appears to have minimal involvement in the onset of periodontitis in SLE patients. Additionally, anti-CCP antibodies are infrequently detected in individuals with SLE (29, 30). Nonetheless, both RA and SLE may overlap, causing a syndrome termed “rhupus” whereby features of both diseases appear in the same patient (31). Furthermore, a positive association between anti-CCP antibodies and erosive arthritis has been reported in rhupus (31). Consequently, while periodontitis might contribute to the onset of RA, a similar association may ostensibly also occur for SLE.

Considering the overlap between RA and SLE, the magnitude of the burden of periodontitis in SLE compared to RA patients has not been explored. Therefore, the aim of this meta-analysis and network meta-analysis (NMA) is to compare: (i) the prevalence of periodontitis in SLE patients compared to versus those with RA and (ii) the odds of developing periodontitis in controls, RA, and SLE to evaluate the magnitude of this comorbidity in SLE.

## 2 Materials and methods

### 2.1 Literature search and study retrieval

This meta-analysis adhered to the guidelines outlined in the Preferred Reporting Items for Systematic Reviews and Meta-Analyses (PRISMA) statement. Two independent investigators (P.R.T. and A.J.L.L.) conducted searches in the PubMed, Medline, Scopus, and EMBASE databases from their inception dates. The searches were repeated just before the final analyses on 9 April 2023. A combination of search terms such as “systemic lupus erythematosus” or “SLE”, “rheumatoid arthritis” or “RA”, “periodontitis” or “chronic periodontitis” or “adult periodontitis” were used (**Supplementary Table 1**). Articles were first screened based on their titles and abstracts by two authors (P.R.T. and A.J.L.L.). Subsequently, P.R.T. and A.J.L.L. independently reviewed the full texts of eligible articles for inclusion. All disagreements during screening or data extraction were resolved were resolved through consensus between the reviewers or by consulting with the senior author (S.H.T.). The study protocol was registered with PROSPERO (CRD42021272876). The Patient, Intervention, Comparison, and Outcomes question is the following: Is there a difference in prevalence or odds of periodontitis in SLE patients compared to RA?

### 2.2 Inclusion criteria

The inclusion criteria were as follows: (i) cross-sectional or case-control study design; (ii) the study reported a quantitative association, i.e., periodontitis events and sample size in RA/SLE and RA/SLE versus controls to calculate event rate and odds ratio (OR), respectively and (iii) the language was limited to English. If the same population was reported in multiple studies, only the most comprehensive study with the largest sample size was included. Patients with periodontitis, RA, and SLE were enrolled in the various studies either based on various definitions, classification criteria, or physician diagnoses. These details are provided in **Table 1** and **Supplementary Table 2**.

**Table 1:** Characteristics of the SLE studies included in the meta-analysis.

Study (1^st^ Author, Year, Reference)	Country/Study Design	Sample Size, n	Type of Control	SLE Classification Criteria	Periodontitis Definition	Immunosuppressants	Age	Female Gender, n (%)	Severity of periodontitis	Quality Assessment
Gofur et al., 2021 (32)	Indonesia/Cross-Sectional	SLE: 61 Control: 61	Healthy	2012 SLICC	PI, GI, CAL, BOP, plaque index, calculus index and numbers of mobility tooth	N.A.	N.A.	SLE: 61 (50) Control: 61 (50)	Reported.	8
Marques et al., 2021 (33)	Brazil/Case Control	SLE: 42 Control: 35	Non-SLE	ACR 1997	Clinically established periodontitis criteria: CAL ≥ 6 mm in at least 2 teeth and 1 or more sites with PD ≥ 5 mm	Reported	Median, IQR Control: 43 (35-56) SLE: 42.5 (33-48)	N.A.	N.A.	7
Pessoa et al., 2019 (34)	Brazil/Case Control	SLE: 60 Control: 31	Healthy	ACR 1997	CDC/AAP	N.A.	Categorised	SLE: 60 (100) Control: 31 (100)	N.A.	5
Mendonça et al., 2019 (35)	Brazil/Case Control	SLE: 70 Control: 70	Non-SLE	N.A.	≥2 interproximal sites with CAL ≥3 mm, and ≥2 interproximal sites with PD ≥4 mm (not on same tooth) or one site with PD ≥5 mm	Prednisolone, antimalarial and immunosuppressant.	Control: 40.9 (+/-14.07) SLE: 37.31 (+/-9.82)	SLE: 63 (90) Control: 56 (77)	N.A.	7
Corrêa et al., 2018 (36)	Brazil/Cross-Sectional	SLE: 75 Control: 78	Non-SLE	N.A.	≥2 interproximal sites with CAL ≥3 mm, and ≥2 interproximal sites with PD ≥4 mm (not on same tooth) or one site with PD ≥5 mm	N.A.	Categorised	SLE: 68 (91) Control: 62 (79)	N.A.	9
Zhang et al., 2017 (37)	China/Case Control	SLE: 108 Control: 108	Healthy	ACR 1997	Periodontal parameters consisted of PI, GI, PPD, CAL, and BOP.	Prednisone, antimalarial, immunosuppressant.	Control: 39.05 (+/-10.27) SLE: 37.48 (+/-9.61)	SLE: 108 (100) Control: 108 (100)	Controls: mild 33; moderate 17; severe 3 SLE: mild 18; moderate 46; severe 26	7
Wu et al., 2017 (38)	Taiwan/Cross-Sectional	SLE: 7,204 Control: 72,040	Non-SLE	ACR 1997	Patients who had one or more outpatient visit before the index date which diagnosed them as having periodontitis (ICD9-CM codes 523.3–523.5), and who were concurrently treated with antibiotics, or dental scaling 3 times per year by certified dentists, were identified as patients with a history of periodontitis	N.A.	Control: 40 (+/-18) SLE: 40 (+/-18)	SLE: 6,199 (86) Control: 61,990 (86)	N.A.	9
Corrêa et al., 2017 (39)	Brazil/Case Control	SLE: 52 Control: 52	Non-SLE	ACR 1997	Periodontal parameters consisted of PI, GI, PPD, CAL and BOP.	N.A.	Categorised	SLE: 25 (48) Control: 22 (42)	N.A.	5
Calderaro et al., 2017 (40)	Brazil/Case Control	SLE: 75 Control: 75	Non-SLE	ACR 1997	No evidence of periodontitis; mild periodontitis ≥2 interproximal sites with CAL ≥3 mm, and ≥2 interproximal sites with PD ≥4 mm (not on the same tooth) or one site with PD ≥5 mm; moderate periodontitis ≥2 interproximal sites with CAL ≥4 mm (not on the same tooth), or ≥2 interproximal sites with PD ≥5 mm (not on same tooth); severe periodontitis: ≥ 2 interproximal sites with CAL ≥ 6 mm (not on same tooth) and ≥1 interproximal site with PD ≥5 mm	Prednisolone, antimalarial, immunosuppressant.	Control: 41 (+/-13.9) SLE: 38 (+/-9.8)	SLE: 68 (91) Control: 58 (77)	Controls: mild 1; moderate 28; severe 13 SLE: mild 2; moderate 36; severe 13	7
Wang et al., 2015 (41)	Taiwan/Case Control	SLE: 53 Control: 56	Healthy	N.A.	≥20% of tooth sites with PD ≥4 mm or CAL ≥4 mm	Immunosuppressants, immunomodulators.	Control: 44.4 SLE: 46.7	SLE: 53 (100) Control: 56 (100)	N.A.	8

SLE, systemic lupus erythematosus; SLICC, Systemic Lupus International Collaborating Clinics Criteria; ACR, American College of Rheumatology; CP, chronic periodontitis; N.A., not available; PI, periodontal index; GI, gingival index; CAL, clinical attachment loss; BOP, bleeding on probing; PD, probing depth; PPD, pocket probing depth; CDC/AAP, Centers for Disease Control and Prevention in partnership with the American Academy of Periodontology.

Data are mean +/- SD or frequency (%), unless otherwise specified.

### 2.3 Exclusion criteria

Articles were excluded based on the following: (i) periodontal parameters were reported but a diagnosis of periodontitis was not made; (ii) diagnoses of RA or SLE were not reported; (iii) participants were enrolled based on a diagnosis of periodontitis as an entry criterion and not that of RA or SLE and (iv) animal studies, case reports and reviews.

### 2.4 Quality assessment

The quality assessment of case-control studies was conducted using the Newcastle–Ottawa Scale (NOS), while cross-sectional studies were evaluated using the modified NOS (42). Studies with NOS scores ≤3, 4–6, and ≥7 were categorized as low, moderate, and high quality, respectively.

### 2.5 Data extraction

The following data were extracted from each included study: (i) study characteristics, including the first author, publication year, region, study design, sample size, RA/SLE classification criteria, periodontitis definition, inclusion and exclusion criteria for cases and controls; (ii) study participant demographics, including mean age, sex, RA/SLE duration, and smoking status; (iii) periodontal measures, including the definition of periodontitis and prevalence of periodontitis and (iv) disease activity and markers, including SLE Disease Activity Index (SLEDAI) for SLE and Disease Activity Score in 28 joints (DAS28) for RA. Due to differences in the definitions of controls across studies, for the NMA, the control populations across the RA and SLE studies are homogenized under the working definition of “absence of known immune-mediated inflammatory disorders and dental diseases”. Thus, this homogenized group consists of healthy, osteoarthritis patients, non-RA and non-SLE controls.

### 2.6 Data synthesis

This study aimed to examine the proportion of periodontitis in patients with RA/SLE and to compare the ORs for periodontitis of RA and SLE with each other and with controls. Pooled proportions were computed using the inverse variance method with the variance-stabilising Freeman-Tukey double arcsine transformation. The confidence intervals (CIs) for individual studies were calculated using the Wilson score CI method with continuity correction. Subgroup analyses were conducted between patients with SLE and those with RA. Differences between pooled prevalence were evaluated using between-subgroup heterogeneity. Meta-regression was attempted using disease activity as a covariate to investigate the association between disease activity and the prevalence of periodontitis. The I^2^ statistic was used to represent between-study heterogeneity, where I^2^ ≤30%, between >30% and ≤50%, between >50% and <75%, and ≥75% were considered to indicate low, moderate, substantial, and considerable heterogeneity, respectively. A NMA was done to indirectly compare if periodontitis is more likely to be associated with RA or SLE. To harmonise the controls from the RA and SLE papers into a single common group, controls were defined as the absence of immune-mediated inflammatory disorders and known dental diseases before study enrollment. The networks were built with controls, RA patients, and SLE patients. As there was no closed loop in the network, inconsistency could not be evaluated in this NMA. Subgroup analyses were conducted to identify the source of heterogeneity as well as to analyze the diversity among different subgroups. Potential publication bias was assessed using a funnel plot and the Egger’s test (43). Further assessment of publication bias was done using Duval and Tweedie trim-and-fill method (44). All analyses were performed using R version 4.1.2, with *metafor* and *netmeta* packages. Statistical significance was set at two-sided p<0.05.

## 3 Results

### 3.1 Characteristics of the selected studies

**Figure 1:**
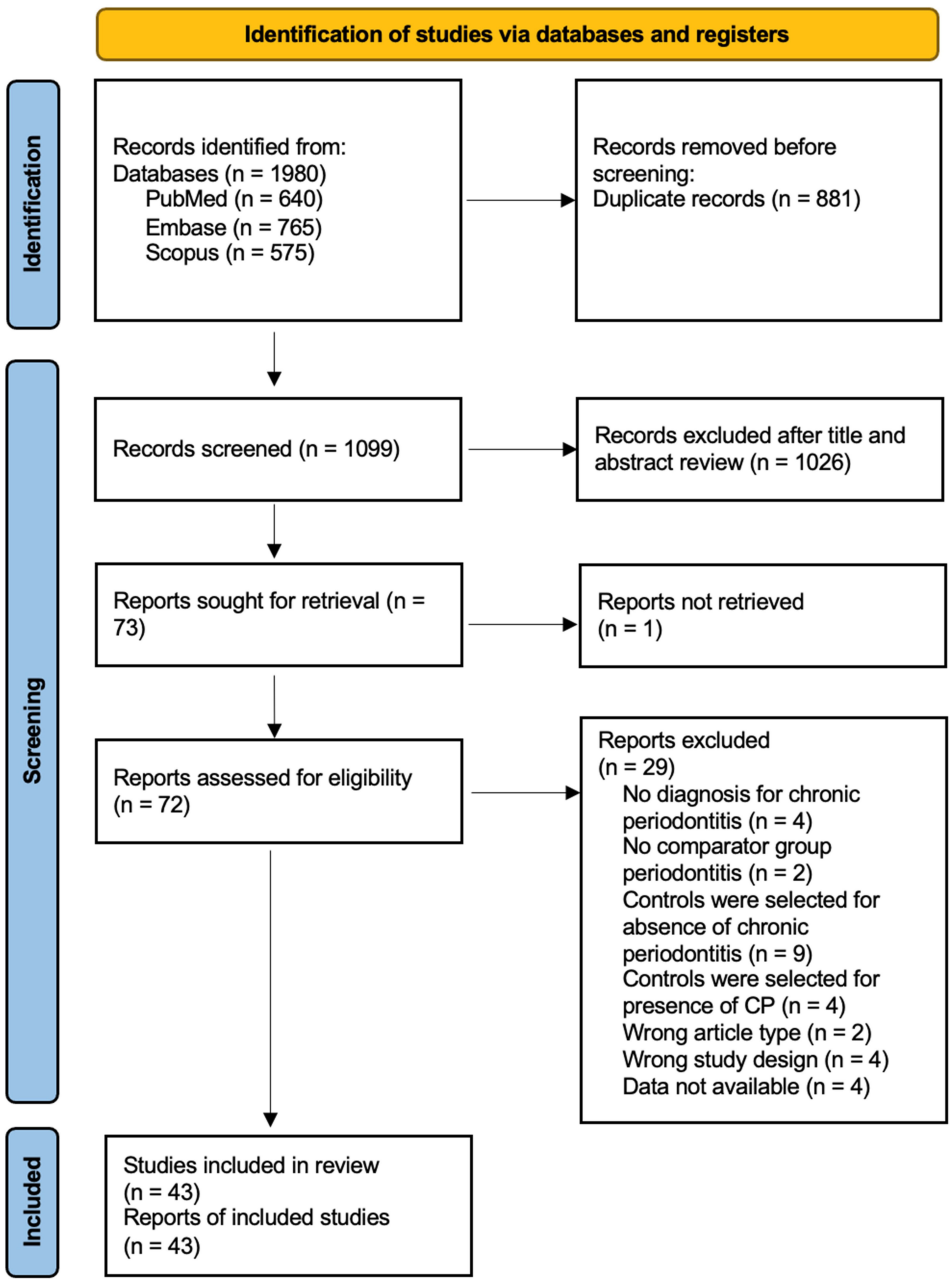
PRISMA flow diagram for RA and SLE studies.

The search strategy yielded 230 and 1,751 potentially relevant SLE and RA studies, respectively. 102 SLE articles and 997 RA articles remained after duplicates were removed. Of the 1099 abstracts screened, 18 SLE and 54 RA studies met the inclusion and exclusion criteria. Their full texts were reviewed. Finally, 10 SLE (32–41) and 33 RA (24, 45–74) studies, i.e., 43 in total, were deemed eligible for the meta-analysis (**Figure 1**, **Table 1**; **Supplementary Table 2**).

### 3.2 Quality appraisal of included studies

Of the 43 studies that underwent quality assessment using the Newcastle-Ottawa checklist, 14 (2 SLE studies and 14 RA studies) obtained a score between 4-6 (moderate quality), while the remaining 29 (8 SLE studies and 21 RA studies) obtained a score of ≥7 (high quality) (**Table 1**; **Supplementary Table 2**). The limitations of the studies regarded as moderate quality were mainly related to the sampling methods.

### 3.3 Periodontitis in RA and SLE

**Figure 2:**
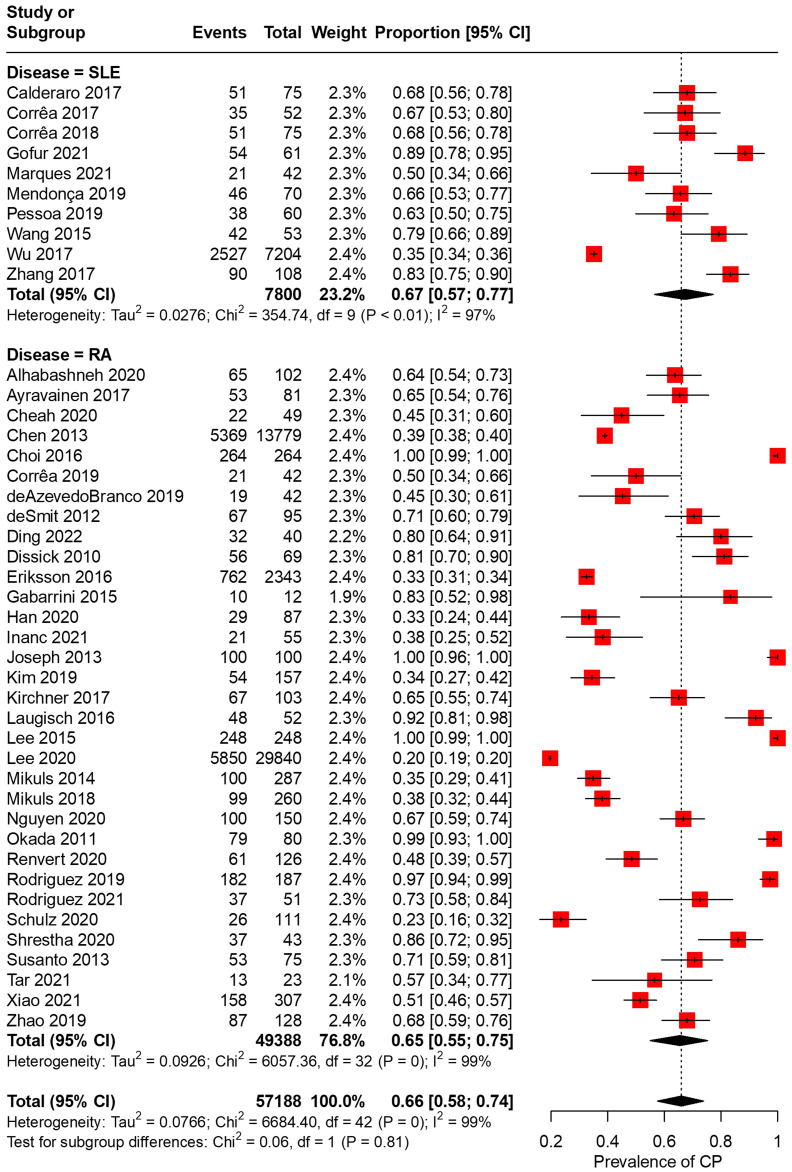
Pooled prevalences of periodontitis in RA and SLE.

**Figure 3:**
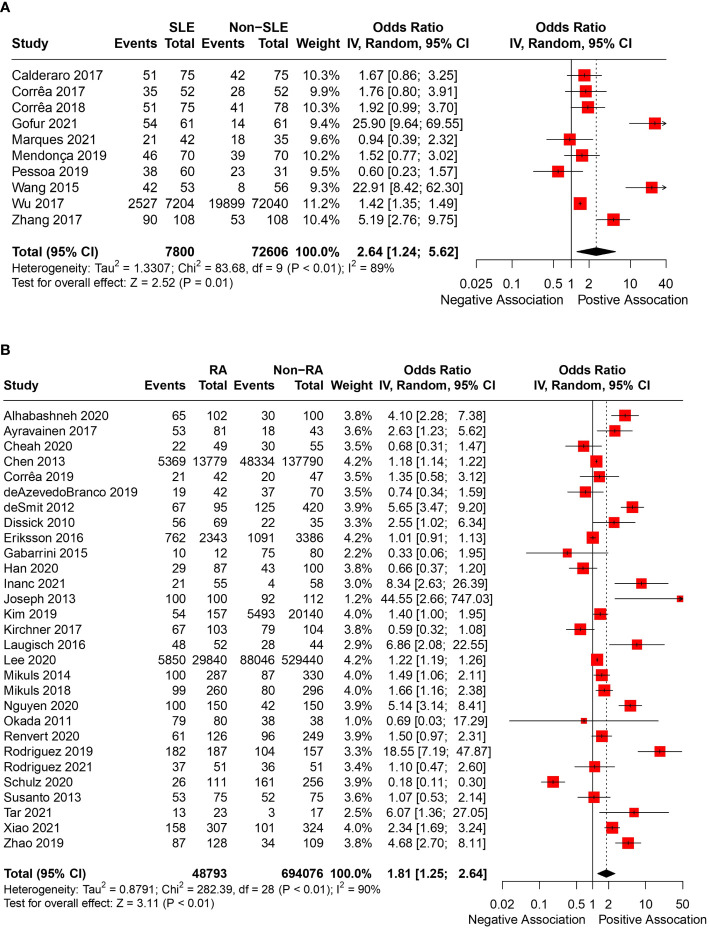
**(A)** Pooled OR of periodontitis in SLE and controls. **(B)** Pooled OR of periodontitis in RA and controls.

**Figure 4:**
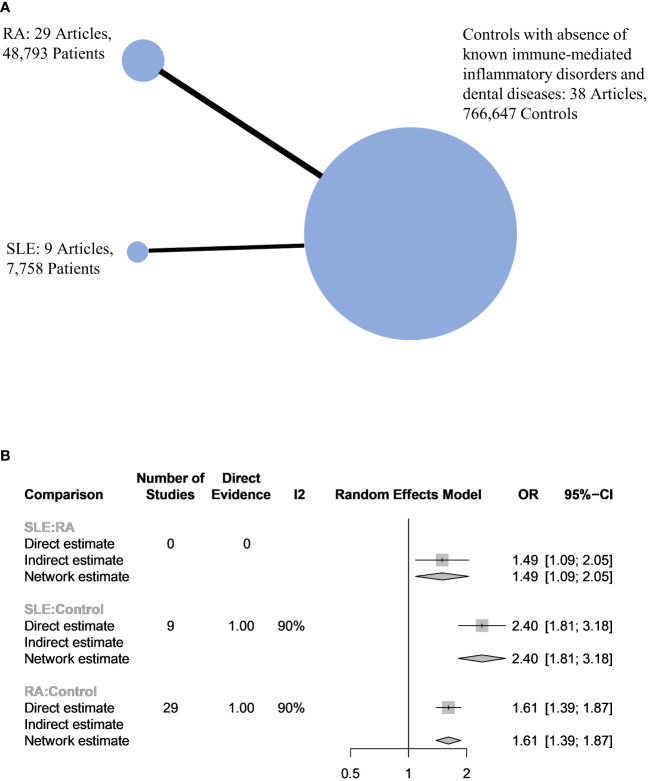
**(A)** Network plot of the 3 populations. The size of the circles is proportional to the number of subjects in each group. The line widths are proportional to the number of studies in the comparison. **(B)** Network meta-analysis between harmonized controls, RA and SLE patients.

In total, 43 articles were incorporated into this meta-analysis. The demographic and clinical characteristics of the included RA and SLE studies are presented in **Supplementary Table 2** and **Table 1**, respectively. A total of 7,800 SLE patients and 49,388 RA patients were studied. Most of the studies originated from Brazil, Taiwan, the United States, Korea, Malaysia, and the Netherlands (in descending order). Ongoing treatment for the underlying rheumatic disease included the use of nonsteroidal anti-inflammatory drugs, corticosteroids, disease-modifying antirheumatic drugs, steroid sparers, and biologics such as tumour necrosis factor-alpha inhibitors, where appropriate. Some studies reported the severity of periodontitis in RA and SLE with variability in the criteria used for mild, moderate, or severe periodontitis ascertainment. The various methods used to ascertain periodontitis are shown in **Supplementary Table 3**. In total, 2,955 SLE patients and 14,189 RA patients were found to have periodontitis. The combined prevalence of periodontitis in both diseases was 66.0% (95% CI 58.0-74.0%). The pooled prevalence of periodontitis in SLE patients (67.0%, 95% CI 57.0-77.0%) was comparable to that of RA (65.0%, 95% CI 55.0-75.0%) with statistically insignificant subgroup effect (p=0.81) (**Figure 2**). The pooled prevalence of periodontitis in SLE controls (45.0%, 95% CI 33.0-57.0%) was slightly lower compared to RA controls (52.0%, 95% CI 41.0-64.0%) (p=0.37) (**Supplementary Figure 1**). Compared to controls, patients with SLE (OR=2.64, 95% CI 1.24-5.62, p<0.01) (**Figure 3A**) and RA (OR=1.81, 95% CI 1.25-2.64, p<0.01) (**Figure 3B**) had significantly greater odds of having periodontitis. 38 articles were included in the NMA. Four articles on RA were excluded because they lacked data on control patients, while one SLE study by Marques et al. was excluded as it enrolled patients with dental diseases as controls (33, 52, 53, 71, 75). **Figure 4A** depicts the network diagram. Indirect comparison through the NMA demonstrated that the odds of having periodontitis in SLE were 1.49 times higher compared to RA (OR=1.49, 95% CI 1.09-2.05, p<0.05), contributed by the lower prevalence of periodontitis in SLE controls relative to RA controls (**Figure 4B**; **Supplementary Figure 1**).

**Supplementary Tables 4**, **5** depict the disease activity of each rheumatic disease and the prevalence/severity of periodontitis if reported. The disease activity indices used were SLEDAI 2000, and DAS28 calculated using erythrocyte sedimentation rate and C-reactive protein (CRP). Owing to the different disease activity scoring systems used and the small number of studies, meta-regression to identify associations between disease activity and periodontitis was not pursued.

### 3.4 Subgroups analysis

To ascertain the primary sources of heterogeneity, we performed a subgroup analysis based on the classification or diagnostic method of periodontitis, study design (cross-sectional or case-control study), publication year grouping, and whether the studies were published before or after June 2018 (1). The pooled prevalence of periodontitis in RA was significantly lower in studies published after June 2018 (**Table 2**).

Table 2Results of subgroups analysis.

**Table:** Prevalence of periodontitis using the same definition.

Definition: CDC/AAP			
Disease group	No. of studies	OR	95% CI
SLE	1	0.63	0.50 – 0.75
RA	4	0.53	0.37 – 0.69
Test for Subgroup difference: p=0.32

**Table:** Prevalence of periodontitis using the same definition.

Definition: CAL of ≥ 6 mm on ≥ 2 teeth, and one or more sites with PD of ≥ 5 mm			
Disease group	No. of studies	OR	95% CI
SLE	1	0.5	0.34 – 0.66
RA	2	0.36	0.32 – 0.40
Test for Subgroup difference: p=0.08

**Table:** Prevalence of periodontitis.

RA			
Study design	No. of studies	Prevalence	95% CI
Cross Sectional	13	0.6792	0.5121 – 0.8260
Case Control	20	0.6387	0.5000 – 0.7668
Test for Subgroup difference: p=0.7033

**Table:** 

SLE			
Study design	No. of studies	Prevalence	95% CI
Cross Sectional	4	0.6838	0.4346 – 0.8878
Case Control	6	0.6746	0.5819 – 0.7610
Test for Subgroup difference: p=0.9391

**Table:** 

RA			
Year group	No. of studies	Prevalence	95% CI
2006 – 2010	1	0.8116	0.7098 – 0.8962
2011 - 2015	10	0.855	0.6676 – 0.9751
2016 - 2020	18	0.5367	0.4240 – 0.6475
2021 - 2023	4	0.5647	0.3867 – 0.7349
Test for Subgroup difference: p=0.0005

**Table:** 

SLE			
Year group	No. of studies	Prevalence	95% CI
2006 – 2010	0	–	–
2011 - 2015	1	0.7925	0.6716 – 0.8923
2016 - 2020	7	0.6431	0.5214 – 0.7562
2021 - 2023	2	0.7147	0.2973 – 0.9872
Test for Subgroup difference: p=0.2137

**Table:** 

RA			
Year group	No. of studies	Prevalence	95% CI
Before June 2018	14	0.7928	0.6315 - 0.9178
After June 2018	19	0.5423	0.4333 - 0.6494
Test for Subgroup difference: p=0.0117

**Table:** 

SLE			
Year group	No. of studies	Prevalence	95% CI
Before June 2018	6	0.6688	0.5192 – 0.8030
After June 2018	4	0.6817	0.5071 - 0.8340
Test for Subgroup difference: p=0.9152

**Table:** Odds of developing periodontitis compared to controls using the same definition of periodontitis.

Definition: CDC/AAP			
Disease group	No. of studies	OR	95% CI
SLE	1	0.6	0.23 – 1.57
RA	4	1.59	0.58 – 4.40
Test for Subgroup difference: p=0.17

**Table:** 

Definition: CAL of ≥ 6 mm on ≥ 2 teeth, and one or more sites with PD of ≥ 5 mm			
Disease group	No. of studies	OR	95% CI
SLE	1	0.94	0.39 – 2.32
RA	2	1.57	1.23 – 2.01
Test for Subgroup difference: p=0.28

**Table:** Odds of developing periodontitis compared to controls.

RA			
Study design	No. of studies	OR	95% CI
Cross Sectional	9	1.2533	1.3479 – 3.7269
Case Control	20	2.2413	0.7781– 2.0186
Test for Subgroup difference: p=0.1021

**Table:** 

SLE			
Study design	No. of studies	OR	95% CI
Cross Sectional	4	5.9819	1.2923 - 27.6886
Case Control	6	1.6191	0.9050 - 2.8967
Test for Subgroup difference: p=0.1181

**Table:** 

RA			
Year group	No. of studies	OR	95% CI
2006 – 2010	1	2.5455	1.0212 – 6.3449
2011 - 2015	8	2.0778	0.9528 – 4.5312
2016 - 2020	17	1.4855	0.9129 – 2.4174
2021 - 2023	3	4.1083	1.6913 – 9.9796
Test for Subgroup difference: p=0.2370

**Table:** 

SLE			
Year group	No. of studies	OR	95% CI
2006 – 2010	0	–	–
2011 - 2015	1	22.9091	8.4240 – 62.3014
2016 - 2020	7	1.736	1.1396 – 2.6446
2021 - 2023	2	4.9125	0.1914 – 126.0651
Test for Subgroup difference: p<0.0001

**Table:** 

RA			
Year group	No. of studies	OR	95% CI
Before June 2018	12	1.7342	1.0334 - 2.9104
After June 2018	17	1.8595	1.1036 - 3.1331
Test for Subgroup difference: p=0.8525

**Table:** 

SLE			
Year group	No. of studies	OR	95% CI
Before June 2018	6	2.9706	1.3316 - 6.6270
After June 2018	4	2.1555	0.4194 - 11.0779
Test for Subgroup difference: p=0.7302			

SLE, systemic lupus erythematosus; RA, rheumatoid arthritis; CI, confidence interval; OR, odds ratio; CDC/AAP, Centers for Disease Control and Prevention (CDC) in collaboration with the American Academy of Periodontology; CAL, clinical attachment level; PD, pocket depth.

### 3.5 Publication bias

**Figure 5:**
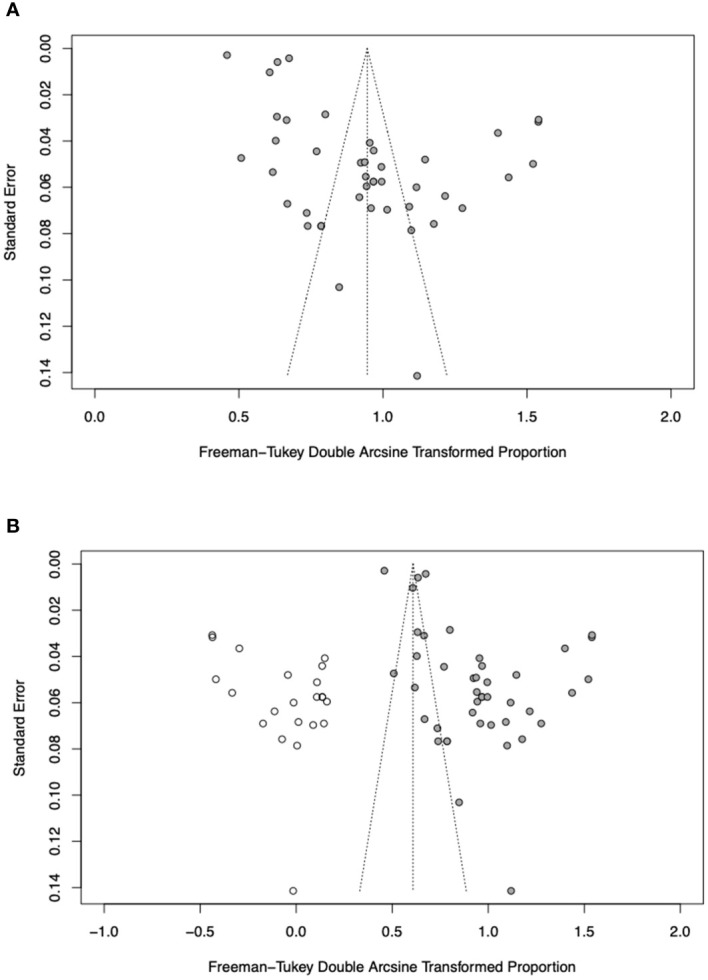
**(A)** Funnel plot of prevalence of periodontitis in RA and SLE patients. **(B)** Filled funnel plot of prevalence of periodontitis in RA and SLE patients. Solid grey circles represent the 41 studies and open circles denote “filled” studies.

There was some asymmetry observed on visual inspection of the funnel plot (**Figure 5A**). Egger’s test indicated substantial evidence of publication bias (p< 0.0001). A sensitivity analysis employing the trim-and-fill method was conducted with 22 imputed studies. The trim-and-fill method yielded a lower pooled prevalence of 32.4% (95% CI, 20.6–45.4%) in SLE and RA patients (**Figure 5B**). This suggests that the true pooled prevalence might be less compared to what was found in this study.

## 4 Discussion

To the best of our knowledge, this meta-analysis and NMA represent the first attempt to compare the prevalence and odds of periodontitis in SLE compared to RA. The principal findings of our meta-analysis and NMA include: (i) the prevalence of periodontitis in patients with SLE was comparable to that of RA, afflicting more than 60% of both patient groups; (ii) both RA and SLE patients exhibited higher odds of having periodontitis compared to controls and (iii) SLE patients were more likely to have periodontitis (1.49-fold higher odds) compared to RA. Similar to previously conducted meta-analyses on periodontitis in patients with RA and SLE, our study showed significantly increased odds of periodontitis in both patient groups compared to controls (9, 10, 46, 76). RA is the most widely recognised rheumatic disease associated with periodontitis, partly because of the key role of citrullination induced by *Porphyromonas gingivalis* and the induction of anti-CCP antibodies that are specific for this disease (22). As a result, the association between RA and periodontitis, as well as dental care considerations specific to RA, have been extensively studied (46, 76, 77). However, the association between SLE and periodontitis is not well recognised by the dental and rheumatology communities (S.H.T., personal communication). Although our study established that there was no statistically significant difference in the prevalence of periodontitis between SLE and RA, it demonstrated that SLE patients have an increased odds of developing periodontitis. Therefore, awareness of the association between SLE and periodontitis is warranted.

The difference in awareness between the associations of periodontitis with RA and SLE may stem from variations in our understanding of their immunopathogenesis. Following the discovery that *Porphyromonas gingivalis* secretes PPAD (22), it was hypothesized that periodontitis may accelerate the process of citrullination within the mouth and lead to increased exposure to citrullinated proteins. Consequently, anti-CCP antibodies are produced, which have been linked to more severe RA and joint destruction (78). Additionally, smoking is known to increase the formation of citrullinated proteins, potentially leading to autoimmunity and the development of RA in susceptible individuals (78). These lines of evidence provided a clear link between periodontitis and RA. While most observational studies do not fully establish this relationship, the axiomatic understanding that periodontitis and RA share a causal relationship may have provided greater awareness of this association. On the other hand, while the possibility of a bidirectional association between periodontitis and SLE has been proposed, the evidence supporting it remains relatively weak (79). Several lines of preclinical evidence explanation have supported the association between periodontitis and SLE. First, increased expression of Toll-like receptors 2 and 4, which are involved in innate immune responses, has been observed in both periodontal disease and SLE (80). Second, the innate immune dysregulation in SLE patients with overactive phagocytic cells leads to elevated production of pro-inflammatory cytokines, such as interleukin (IL)-1β and IL-18, which are implicated in the pathogenesis of periodontitis (81, 82). Third, in patients with periodontitis, B lymphocytes and plasma cells are increased in the periodontal tissues. These cells are also the adaptive immune cells implicated in the immunopathogenesis of SLE (82, 83). Fourth, periodontal bacteria may stimulate antiphospholipid antibody production through the process of molecular mimicry between bacterial peptides and β2-glycoprotein I (41). Fifth, a recent Mendelian randomization analysis indicated that periodontitis is associated with a weak causal association with SLE (84). Lastly, immunosuppression using corticosteroids and steroid sparers leads to a reduction in host immunity and consequently repeated oral infections (85, 86).

The most common oral manifestation observed in SLE is painless ulcers typically found in the lip and buccal mucosa (87). While oral manifestations of SLE have been estimated to vary from 9-45% (87), owing to the lack of awareness that periodontitis may be associated with SLE, its suspicion may not be entertained, resulting in delayed diagnosis and intervention (88). Consequently, this delay contributes to poorer dental outcomes, tooth loss, and diminished quality of life among SLE patients (4–6). Despite the burden of periodontitis in SLE, there is a dearth of literature on the awareness of medical professionals on this comorbidity. In contrast, the level of awareness regarding the relationship between periodontitis and RA has been documented in medical literature. Afilal et al. conducted a cross-sectional survey revealing that only 6% of rheumatologists routinely examined the oral cavity, while 11% acknowledged the negative impact of poor oral hygiene on RA, and 10% recommended dental consultation for RA patients (89). Similarly, Nazir et al. found that 36.2% of dentists were aware of the association between periodontal disease and rheumatoid arthritis (90). There is a pressing need for studies to explore the level of awareness regarding the association between SLE and periodontitis within the rheumatology and dental communities.

Numerous studies have demonstrated an association between disease activity in RA/SLE and the severity of periodontitis. RA patients with periodontitis categorized as level 0 or 1 exhibited significantly lower mean DAS28-CRP compared to those with level 2 periodontitis (62). Similarly, SLE patients with higher SLEDAI scores were shown to have more severe periodontal disease (91, 92). Furthermore, a randomised controlled trial by Fabbri et al. found that treatment for periodontitis led to a notable reduction in SLEDAI scores (93). Within 3 months of treatment initiation, a notable decrease in both SLE disease activity and periodontal disease parameters was observed. In contrast, the group without treatment had persistent SLE disease activity and half of the periodontal disease parameters unchanged from baseline (93). Hence, these findings underscore the importance of raising awareness among clinicians about the association between SLE and periodontitis, as managing this modifiable comorbidity might help to modulate SLE disease activity.

The similar prevalence but increased odds of periodontitis for SLE compared to RA in our analysis deserves discussion. One possible explanation is that SLE patients included in the NMA had relatively higher disease activity compared to RA and therefore had more cases of periodontitis as a result of the bidirectional association (79). In addition, the lower pooled prevalences of periodontitis in the controls of SLE (45.0%, 95% CI 33.0-57.0%) compared to RA (52.0%, 95% CI 41.0-64.0%) (**Supplementary Figure 1**) would have contributed to an increased odds of periodontitis for SLE compared to RA.

Rheumatoid arthritis and SLE share several risk-associated loci (e.g., *HLA-DRB1*, *BLK*, *UBE2L3*, *PTPN22*, *STAT4*, *TNFAIP3*, *FCGR2A*, *PRDM1*, *IRF5*, *PXK* and *COG6*) and are characterized by the presence of autoantibodies that recognize self-antigens (15, 16, 31, 94). Environmental triggers such as tobacco smoking have also been described in both diseases, although the prevalence of smoking in both RA and SLE is not well described in epidemiological studies (15, 16). Both diseases, however, differ in many aspects such as immunopathogenesis whereby type I interferons in response to viral factors and tumor necrosis factor in relation to microbiota are operational in SLE and RA, respectively (15, 16). Specific class II major histocompatibility molecules contain the shared epitope, a specific amino acid motif associated with the risk of developing RA (15). This epitope facilitates the presentation of arthritogenic peptides to CD4^+^ T cells, particularly those containing citrulline, thereby promoting the development of anti-CCP antibodies (31). The interaction between the shared epitope and smoking has been extensively studied and its interplay with *Porphyromonas gingivalis* in arthritis-prone B6.DR1 mice leading to increased anti-CCP production makes periodontitis an even more compelling risk factor for RA (95). RA is considered a continuum that begins with a high-risk or susceptibility state influenced by genetic factors and progresses through preclinical, early, and established disease, where environmental factors contribute to the inflammatory and destructive synovial response (15). Preclinical and early RA are different from rhupus, a disease with features that overlap between RA and SLE (31). The progression of arthritis in rhupus mirrors a pattern similar to RA, potentially advancing to typical inflammatory erosions. However, the SLE-related aspects in rhupus tend to be milder, primarily manifesting as hematological and mucocutaneous involvement. (31).

This study has several limitations that should be considered. First, a high heterogeneity was observed. However, in this analysis, heterogeneity was estimated from the I^2^ statistic and it is common for proportional meta-analyses to have a high I^2^, possibly because of the nature of the proportional data (96). The high heterogeneity could also be attributed to variability in the case definition and classification of periodontitis (1, 97). Consistent case definition and classification of periodontitis would contribute to reduced heterogeneity. Of note, prevalence of periodontitis decreased in RA patients after the 2017 World Workshop Classification of Periodontal and Peri-implant Diseases and Conditions was published (**Table 2**), which may reflect reduced heterogeneity in the ascertainment of this odontogenic infection (1). The other possibility is the improvement of RA treatment over time resulting in decreased periodontitis due to a bidirectional effect. Diagnostic criteria for RA and SLE have not been endorsed by major rheumatology societies and only classification criteria have been developed to recruit homogenous populations for research (15, 16). These classification criteria are not intended for use in clinical practice as the basis for establishing the diagnoses of RA and SLE. As such, some RA and SLE patients in the studies that did not specify the classification criteria used were diagnosed clinically. In addition, specific endotypes of RA (e.g., seropositive and seronegative) and SLE (e.g., organ-dominant, lupus with antiphospholipid syndrome and Sjögren’s syndrome) were not reported in the studies (98, 99). Hence, transparent reporting of the classification criteria used for classification and any underlying endotypes would have facilitated a more accurate interpretation of our results. Second, evidence of publication bias was detected, suggesting an overestimation of prevalence in our meta-analysis due to potential unpublished studies. Third, due to the many different scoring systems used for disease activity and the limited number of studies, we were unable to perform a meta-regression to explore the relationship between rheumatological disease activity and the prevalence of periodontitis. Fourth, smoking and the use of immunosuppressants may be confounding factors that could contribute to the development of periodontitis. However, these confounding factors were not assessed in many of the included studies and should be analyzed in detail for future work. Lastly, most of the included studies were cross-sectional in design, limiting their ability to establish a causal relationship between periodontitis and SLE. Future well-designed longitudinal, case-control or cohort studies are needed to explore this relationship and determine if temporal and causal links exist between these two conditions (100). By addressing these limitations, future researchers can help to advance the understanding between periodontitis and rheumatic diseases by improving the quality of the research in this field.

This meta-analysis and NMA established that SLE patients have a significantly higher likelihood of experiencing periodontitis (1.49-fold higher odds) compared to RA, although no significant difference in the prevalence of periodontitis was found between RA and SLE. Considering that RA is traditionally linked with periodontal disease, the elevated odds of periodontitis in SLE are striking. Irrespective of the highlighted limitations, especially that of variations in periodontitis assessment criteria among the studies, these results underscore the importance of addressing the dental health needs of SLE patients. Future research should explore the extent of awareness regarding the association between SLE and periodontitis within the dental and rheumatology communities. Enhancing awareness among healthcare providers and enhancing educational efforts in both disciplines will be crucial in alleviating the considerable disease burden of periodontitis in SLE. Further clinical and translational research is warranted to advance the understanding of periodontitis and its broad impact on SLE immunopathogenesis and disease activity.

## Data availability statement

The original contributions presented in the study are included in the article/**Supplementary Material**. Further inquiries can be directed to the corresponding author.

## Author contributions

PT: Writing – original draft, Writing – review & editing, Data curation, Formal analysis, Visualization. AL: Data curation, Formal analysis, Visualization, Writing – original draft, Writing – review & editing. JZ: Formal analysis, Methodology, Writing – original draft, Writing – review & editing. YC: Methodology, Writing – original draft, Writing – review & editing. JF: Conceptualization, Writing – original draft, Writing – review & editing. MM: Conceptualization, Supervision, Writing – original draft, Writing – review & editing. ST: Conceptualization, Methodology, Project administration, Supervision, Writing – original draft, Writing – review & editing.

## References

[1] Tonetti MS Greenwell H Kornman KS . Staging and grading of periodontitis: Framework and proposal of a new classification and case definition. J Periodontol. (2018) 89 Suppl 1:S159–s172. doi: 10.1002/JPER.18-0006 29926952

[2] Könönen E Gursoy M Gursoy UK . Periodontitis: A multifaceted disease of tooth-supporting tissues. J Clin Med. (2019) 8. doi: 10.3390/jcm8081135 PMC6723779 31370168

[3] Van Dyke TE Bartold PM Reynolds EC . The nexus between periodontal inflammation and dysbiosis. Front Immunol. (2020) 11:511. doi: 10.3389/fimmu.2020.00511 32296429 PMC7136396

[4] Tonetti MS Jepsen S Jin L Otomo-Corgel J . Impact of the global burden of periodontal diseases on health, nutrition and wellbeing of mankind: A call for global action. J Clin Periodontol. (2017) 44:456–62. doi: 10.1111/jcpe.12732 28419559

[5] Reynolds I Duane B . Periodontal disease has an impact on patients' quality of life. Evid Based Dent. (2018) 19:14–5. doi: 10.1038/sj.ebd.6401287 29568030

[6] Nazir M Al-Ansari A Al-Khalifa K Alhareky M Gaffar B Almas K . Global prevalence of periodontal disease and lack of its surveillance. ScientificWorldJournal. (2020) 2020. doi: 10.1155/2020/2146160 PMC7275199 32549797

[7] Winning L Linden GJ . Periodontitis and systemic disease. BDJ Team. (2015) 2:15163. doi: 10.1038/bdjteam.2015.163 PMC5332497 28303212

[8] Global, regional, and national incidence, prevalence, and years lived with disability for 328 diseases and injuries for 195 countries 1990-2016: a systematic analysis for the Global Burden of Disease Study 2016. Lancet. (2017) 390:1211–59. doi: 10.1016/s0140-6736(17)32154-2 PMC5605509 28919117

[9] Rutter-Locher Z Smith TO Giles I Sofat N . Association between systemic lupus erythematosus and periodontitis: A systematic review and meta-analysis. Front Immunol. (2017) 8:1295. doi: 10.3389/fimmu.2017.01295 29089946 PMC5650969

[10] Zhong HJ Xie HX Luo XM Zhang EH . Association between periodontitis and systemic lupus erythematosus: a meta-analysis. Lupus. (2020) 29:1189–97. doi: 10.1177/0961203320938447 32635879

[11] Li Y Guo R Oduro PK Sun T Chen H Yi Y . The relationship between porphyromonas gingivalis and rheumatoid arthritis: A meta-analysis. Front Cell Infect Microbiol. (2022) 12:956417. doi: 10.3389/fcimb.2022.956417 35923803 PMC9340274

[12] Aljohani K Alzahrani AS . Awareness among medical and dental students regarding the relationship between periodontal and systemic conditions. Int J Pharm Res Allied Sci. (2017) 6:61–72.

[13] Park H Bourla AB Kastner DL Colbert RA Siegel RM . Lighting the fires within: the cell biology of autoinflammatory diseases. Nat Rev Immunol. (2012) 12:570–80. doi: 10.1038/nri3261 PMC4165575 22828911

[14] Cross M Smith E Hoy D Carmona L Wolfe F Vos T . The global burden of rheumatoid arthritis: estimates from the global burden of disease 2010 study. Ann Rheum Dis. (2014) 73:1316–22. doi: 10.1136/annrheumdis-2013-204627 24550173

[15] Smolen JS Aletaha D Barton A Burmester GR Emery P Firestein GS . Rheumatoid arthritis. Nat Rev Dis Primers. (2018) 4:18001. doi: 10.1038/nrdp.2018.1 29417936

[16] Kaul A Gordon C Crow MK Touma Z Urowitz MB Van Vollenhoven R . Systemic lupus erythematosus. Nat Rev Dis Primers. (2016) 2:16039. doi: 10.1038/nrdp.2016.39 27306639

[17] Lewis MJ Jawad AS . The effect of ethnicity and genetic ancestry on the epidemiology, clinical features and outcome of systemic lupus erythematosus. Rheumatology. (2017) 56:i67–77. doi: 10.1093/rheumatology/kew399 27940583

[18] Ballestar E Li T . New insights into the epigenetics of inflammatory rheumatic diseases. Nat Rev Rheumatol. (2017) 13:593–605. doi: 10.1038/nrrheum.2017.147 28905855

[19] Deane KD Demoruelle MK Kelmenson LB Kuhn KA Norris JM Holers VM . Genetic and environmental risk factors for rheumatoid arthritis. Best Pract Res Clin Rheumatol. (2017) 31:3–18. doi: 10.1016/j.berh.2017.08.003 29221595 PMC5726551

[20] Moulton VR Suarez-Fueyo A Meidan E Li H Mizui M Tsokos GC . Pathogenesis of human systemic lupus erythematosus: A cellular perspective. Trends Mol Med. (2017) 23:615–35. doi: 10.1016/j.molmed.2017.05.006 PMC5650102 28623084

[21] Cusick MF Libbey JE Fujinami RS . Molecular mimicry as a mechanism of autoimmune disease. Clin Rev Allergy Immunol. (2012) 42:102–11. doi: 10.1007/s12016-011-8294-7 PMC3266166 22095454

[22] Olsen I Singhrao SK Potempa J . Citrullination as a plausible link to periodontitis, rheumatoid arthritis, atherosclerosis and Alzheimer's disease. J Oral Microbiol. (2018) 10:1487742. doi: 10.1080/20002297.2018.1487742 29963294 PMC6022223

[23] Koziel J Mydel P Potempa J . The link between periodontal disease and rheumatoid arthritis: an updated review. Curr Rheumatol Rep. (2014) 16:408. doi: 10.1007/s11926-014-0408-9 24458478 PMC3930831

[24] Dissick A Redman RS Jones M Rangan BV Reimold A Griffiths GR . Association of periodontitis with rheumatoid arthritis: a pilot study. J Periodontol. (2010) 81:223–30. doi: 10.1902/jop.2009.090309 20151800

[25] Eezammuddeen NN Vaithilingam RD Hassan NHM . Influence of periodontitis on levels of autoantibodies in rheumatoid arthritis patients: A systematic review. J Periodontal Res. (2023) 58:29–42. doi: 10.1111/jre.13065 36317493

[26] Zhang J Xu C Gao L Zhang D Li C Liu J . Influence of anti-rheumatic agents on the periodontal condition of patients with rheumatoid arthritis and periodontitis: A systematic review and meta-analysis. J Periodontal Res. (2021) 56:1099–115. doi: 10.1111/jre.12925 34514591

[27] Rutter-Locher Z Smith TO Giles I Sofat N . Association between systemic lupus erythematosus and periodontitis: A systematic review and meta-analysis. Front Immunol. (2017) 8. doi: 10.3389/fimmu.2017.01295 PMC5650969 29089946

[28] Hussain SB Leira Y Zehra SA Botelho J MaChado V Ciurtin C . Periodontitis and Systemic Lupus Erythematosus: A systematic review and meta-analysis. J Periodontal Res. (2022) 57:1–10. doi: 10.1111/jre.12936 34608627

[29] Amezcua-Guerra LM Springall R Marquez-Velasco R Gómez-García L Vargas A Bojalil R . Presence of antibodies against cyclic citrullinated peptides in patients with 'rhupus': a cross-sectional study. Arthritis Res Ther. (2006) 8:R144. doi: 10.1186/ar2036 16934155 PMC1779435

[30] Martínez REM Herrera JLA Pérez RAD Mendoza CA Manrique SIR . Frequency of Porphyromonas gingivalis and fimA genotypes in patients with periodontitis and systemic lupus erythematosus. Lupus. (2021) 30:80–5. doi: 10.1177/0961203320969983 33115372

[31] Antonini L Le Mauff B Marcelli C Aouba A De Boysson H . Rhupus: a systematic literature review. Autoimmun Rev. (2020) 19:102612. doi: 10.1016/j.autrev.2020.102612 32668290

[32] Gofur NRP Handono K Nurdiana N Kalim H . Periodontal comparison on systemic lupus erythematosus patients and healthy subjects: A cross-sectional study. Pesqui Bras Odontopediatria Clín Integr. (2021) 21. doi: 10.1590/pboci.2021.108

[33] Marques CPC Rodrigues VP De Carvalho LC Nichilatti LP Franco MM Patrício FJB . Expression of Toll-like receptors 2 and 4 in the saliva of patients with systemic lupus erythematosus and chronic periodontitis. Clin Rheumatol. (2021) 40:2727–34. doi: 10.1007/s10067-020-05560-z 33570702

[34] Pessoa L Aleti G Choudhury S Nguyen D Yaskell T Zhang Y . Host-microbial interactions in systemic lupus erythematosus and periodontitis. Front Immunol. (2019) 10:2602. doi: 10.3389/fimmu.2019.02602 31781106 PMC6861327

[35] Mendonça SMS Corrêa JD Souza AF Travassos DV Calderaro DC Rocha NP . Immunological signatures in saliva of systemic lupus erythematosus patients: influence of periodontal condition. Clin Exp Rheumatol. (2019) 37:208–14. 30148445

[36] Corrêa JD Branco LGA Calderaro DC Mendonça SMS Travassos DV Ferreira GA . Impact of systemic lupus erythematosus on oral health-related quality of life. Lupus. (2018) 27:283–9. doi: 10.1177/0961203317719147 28679308

[37] Zhang Q Zhang X Feng G Fu T Yin R Zhang L . Periodontal disease in Chinese patients with systemic lupus erythematosus. Rheumatol Int. (2017) 37:1373–9. doi: 10.1007/s00296-017-3759-5 28631047

[38] Wu YD Lin CH Chao WC Liao TL Chen DY Chen HH . Association between a history of periodontitis and the risk of systemic lupus erythematosus in Taiwan: A nationwide, population-based, case-control study. PloS One. (2017) 12:e0187075. doi: 10.1371/journal.pone.0187075 29059229 PMC5653351

[39] Corrêa JD Calderaro DC Ferreira GA Mendonça SM Fernandes GR Xiao E . Subgingival microbiota dysbiosis in systemic lupus erythematosus: association with periodontal status. Microbiome. (2017) 5:34. doi: 10.1186/s40168-017-0252-z 28320468 PMC5359961

[40] Calderaro DC Ferreira GA Corrêa JD Mendonça SM Silva TA Costa FO . Is chronic periodontitis premature in systemic lupus erythematosus patients? Clin Rheumatol. (2017) 36:713–8. doi: 10.1007/s10067-016-3385-8 27557901

[41] Wang CY Chyuan IT Wang YL Kuo MY Chang CW Wu KJ . β2-glycoprotein I-dependent anti-cardiolipin antibodies associated with periodontitis in patients with systemic lupus erythematosus. J Periodontol. (2015) 86:995–1004. doi: 10.1902/jop.2015.140664 25817824

[42] Wells G Shea B O'connell D Peterson J Welch V Losos M . The Newcastle–Ottawa Scale (NOS) for assessing the quality of nonrandomized studies in meta-analyses. (2009). Available at: https://www.ohri.ca/programs/clinical_epidemiology/oxford.asp.

[43] Egger M Davey Smith G Schneider M Minder C . Bias in meta-analysis detected by a simple, graphical test. Bmj. (1997) 315:629–34. doi: 10.1136/bmj.315.7109.629 PMC2127453 9310563

[44] Duval S Tweedie R . Trim and fill: A simple funnel-plot-based method of testing and adjusting for publication bias in meta-analysis. Biometrics. (2000) 56:455–63. doi: 10.1111/j.0006-341X.2000.00455.x 10877304

[45] Okada M Kobayashi T Ito S Yokoyama T Komatsu Y Abe A . Antibody responses to periodontopathic bacteria in relation to rheumatoid arthritis in Japanese adults. J Periodontol. (2011) 82:1433–41. doi: 10.1902/jop.2011.110020 21342003

[46] De Smit M Westra J Vissink A Doornbos-Van Der Meer B Brouwer E Van Winkelhoff AJ . Periodontitis in established rheumatoid arthritis patients: a cross-sectional clinical, microbiological and serological study. Arthritis Res Ther. (2012) 14:R222. doi: 10.1186/ar4061 23075462 PMC3580533

[47] Chen HH Huang N Chen YM Chen TJ Chou P Lee YL . Association between a history of periodontitis and the risk of rheumatoid arthritis: a nationwide, population-based, case-control study. Ann Rheum Dis. (2013) 72:1206–11. doi: 10.1136/annrheumdis-2012-201593 22941768

[48] Joseph R Rajappan S Nath SG Paul BJ . Association between chronic periodontitis and rheumatoid arthritis: a hospital-based case-control study. Rheumatol Int. (2013) 33:103–9. doi: 10.1007/s00296-011-2284-1 22228465

[49] Susanto H Nesse W Kertia N Soeroso J Huijser Van Reenen Y Hoedemaker E . Prevalence and severity of periodontitis in Indonesian patients with rheumatoid arthritis. J Periodontol. (2013) 84:1067–74. doi: 10.1902/jop.2012.110321 23075431

[50] Mikuls TR Payne JB Yu F Thiele GM Reynolds RJ Cannon GW . Periodontitis and Porphyromonas gingivalis in patients with rheumatoid arthritis. Arthritis Rheumatol. (2014) 66:1090–100. doi: 10.1002/art.38348 PMC4115329 24782175

[51] Gabarrini G De Smit M Westra J Brouwer E Vissink A Zhou K . The peptidylarginine deiminase gene is a conserved feature of Porphyromonas gingivalis. Sci Rep. (2015) 5:13936. doi: 10.1038/srep13936 26403779 PMC4585897

[52] Lee JY Choi IA Kim JH Kim KH Lee EY Lee EB . Association between anti-Porphyromonas gingivalis or anti-α-enolase antibody and severity of periodontitis or rheumatoid arthritis (RA) disease activity in RA. BMC Musculoskelet Disord. (2015) 16:190. doi: 10.1186/s12891-015-0647-6 26265263 PMC4542108

[53] Choi IA Kim JH Kim YM Lee JY Kim KH Lee EY . Periodontitis is associated with rheumatoid arthritis: a study with longstanding rheumatoid arthritis patients in Korea. Korean J Intern Med. (2016) 31:977–86. doi: 10.3904/kjim.2015.202 PMC5016284 27017391

[54] Eriksson K Nise L Kats A Luttropp E Catrina AI Askling J . Prevalence of periodontitis in patients with established rheumatoid arthritis: A swedish population based case-control study. PloS One. (2016) 11:e0155956. doi: 10.1371/journal.pone.0155956 27203435 PMC4874595

[55] Laugisch O Wong A Sroka A Kantyka T Koziel J Neuhaus K . Citrullination in the periodontium–a possible link between periodontitis and rheumatoid arthritis. Clin Oral Investig. (2016) 20:675–83. doi: 10.1007/s00784-015-1556-7 PMC5146953 26264638

[56] Äyräväinen L Leirisalo-Repo M Kuuliala A Ahola K Koivuniemi R Meurman JH . Periodontitis in early and chronic rheumatoid arthritis: a prospective follow-up study in Finnish population. BMJ Open. (2017) 7:e011916. doi: 10.1136/bmjopen-2016-011916 PMC5293865 28143836

[57] Kirchner A Jäger J Krohn-Grimberghe B Patschan S Kottmann T Schmalz G . Active matrix metalloproteinase-8 and periodontal bacteria depending on periodontal status in patients with rheumatoid arthritis. J Periodontal Res. (2017) 52:745–54. doi: 10.1111/jre.12443 28321852

[58] Mikuls TR Walker C Qiu F Yu F Thiele GM Alfant B . The subgingival microbiome in patients with established rheumatoid arthritis. Rheumatol (Oxford). (2018) 57:1162–72. doi: 10.1093/rheumatology/key052 29562298

[59] Corrêa JD Fernandes GR Calderaro DC Mendonça SMS Silva JM Albiero ML . Oral microbial dysbiosis linked to worsened periodontal condition in rheumatoid arthritis patients. Sci Rep. (2019) 9:8379. doi: 10.1038/s41598-019-44674-6 31182740 PMC6557833

[60] De Azevedo Branco LG Oliveira SR Corrêa JD Calderaro DC Mendonça SMS De Queiroz Cunha F . Oral health-related quality of life among individuals with rheumatoid arthritis. Clin Rheumatol. (2019) 38:2433–41. doi: 10.1007/s10067-019-04555-9 31004305

[61] Kim JW Park JB Yim HW Lee J Kwok SK Ju JH . Rheumatoid arthritis is associated with early tooth loss: results from Korea National Health and Nutrition Examination Survey V to VI. Korean J Intern Med. (2019) 34:1381–91. doi: 10.3904/kjim.2018.093 PMC6823554 30257550

[62] Rodríguez-Lozano B González-Febles J Garnier-Rodríguez JL Dadlani S Bustabad-Reyes S Sanz M . Association between severity of periodontitis and clinical activity in rheumatoid arthritis patients: a case-control study. Arthritis Res Ther. (2019) 21:27. doi: 10.1186/s13075-019-1808-z 30658685 PMC6339403

[63] Zhao R Gu C Zhang Q Zhou W Feng G Feng X . Periodontal disease in Chinese patients with rheumatoid arthritis: A case-control study. Oral Dis. (2019) 25:2003–9. doi: 10.1111/odi.13176 31411781

[64] Alhabashneh R Alawneh K Alshami R Al Naji K . Rheumatoid arthritis and periodontitis: a Jordanian case-control study. J Public Health. (2020) 28:547–54. doi: 10.1007/s10389-019-01073-5

[65] Cheah CW Al-Maleki AR Vadivelu J Danaee M Sockalingam S Baharuddin NA . Salivary and serum cathelicidin LL-37 levels in subjects with rheumatoid arthritis and chronic periodontitis. Int J Rheumatic Dis. (2020) 23:1344–52. doi: 10.1111/1756-185X.13919 32743970

[66] Han PSH Saub R Baharuddin NA Sockalingam S Bartold PM Vaithilingam RD . Impact of periodontitis on quality of life among subjects with rheumatoid arthritis: a cross sectional study. BMC Oral Health. (2020) 20:332. doi: 10.1186/s12903-020-01275-4 33225923 PMC7682007

[67] Lee KH Choi YY . Rheumatoid arthritis and periodontitis in adults: Using the Korean National Health Insurance Service-National Sample Cohort. J Periodontol. (2020) 91:1186–93. doi: 10.1002/JPER.19-0311 31984496

[68] Nguyen VB Nguyen TT Huynh NC Le TA Hoang HT . Relationship between periodontitis and rheumatoid arthritis in Vietnamese patients. Acta Odontol Scand. (2020) 78:522–8. doi: 10.1080/00016357.2020.1747635 32238080

[69] Renvert S Berglund JS Persson GR Söderlin MK . The association between rheumatoid arthritis and periodontal disease in a population-based cross-sectional case-control study. BMC Rheumatol. (2020) 4:31. doi: 10.1186/s41927-020-00129-4 32699831 PMC7370413

[70] Schulz S Zimmer P Pütz N Jurianz E Schaller H-G Reichert S . rs2476601 in PTPN22 gene in rheumatoid arthritis and periodontitis—a possible interface? J Trans Med. (2020) 18:389. doi: 10.1186/s12967-020-02548-w PMC7559817 33059697

[71] Shrestha S Pradhan S Adhikari B . Prevalence of periodontitis among rheumatoid arthritis patients attending tertiary hospital in Nepal. J Nepal Health Res Counc. (2020) 17:543–7. doi: 10.33314/jnhrc.v17i4 32001864

[72] Inanc N Mumcu G Can M Yay M Silbereisen A Manoil D . Elevated serum TREM-1 is associated with periodontitis and disease activity in rheumatoid arthritis. Sci Rep. (2021) 11:2888. doi: 10.1038/s41598-021-82335-9 33536478 PMC7859204

[73] Rodríguez J Lafaurie GI Bautista-Molano W Chila-Moreno L Bello-Gualtero JM Romero-Sánchez C . Adipokines and periodontal markers as risk indicators of early rheumatoid arthritis: a cross-sectional study. Clin Oral Investig. (2021) 25:1685–95. doi: 10.1007/s00784-020-03469-0 32740810

[74] Tar I Csősz É. Végh E Lundberg K Kharlamova N Soós B . Salivary citrullinated proteins in rheumatoid arthritis and associated periodontal disease. Sci Rep. (2021) 11:13525. doi: 10.1038/s41598-021-93008-y 34188155 PMC8241986

[75] Ding N Luo M Wen YH Li RY Bao QY . The effects of non-surgical periodontitis therapy on the clinical features and serological parameters of patients suffering from rheumatoid arthritis as well as chronic periodontitis. J Inflammation Res. (2022) 15:177–85. doi: 10.2147/JIR.S326896 PMC8760992 35046692

[76] Mercado FB Marshall RI Klestov AC Bartold PM . Relationship between rheumatoid arthritis and periodontitis. J Periodontol. (2001) 72:779–87. doi: 10.1902/jop.2001.72.6.779 11453241

[77] Treister N Glick M . Rheumatoid arthritis: a review and suggested dental care considerations. J Am Dent Assoc. (1999) 130:689–98. doi: 10.14219/jada.archive.1999.0279 10332134

[78] Alsalahy MM Nasser HS Hashem MM Elsayed SM . Effect of tobacco smoking on tissue protein citrullination and disease progression in patients with rheumatoid arthritis. Saudi Pharm J. (2010) 18:75–80. doi: 10.1016/j.jsps.2010.02.002 23960723 PMC3731012

[79] Sojod B Pidorodeski Nagano C Garcia Lopez GM Zalcberg A Dridi SM Anagnostou F . Systemic lupus erythematosus and periodontal disease: A complex clinical and biological interplay. J Clin Med. (2021) 10. doi: 10.3390/jcm10091957 PMC8125164 34063235

[80] Marques CP Maor Y De Andrade MS Rodrigues VP Benatti BB . Possible evidence of systemic lupus erythematosus and periodontal disease association mediated by Toll-like receptors 2 and 4. Clin Exp Immunol. (2016) 183:187–92. doi: 10.1111/cei.12708 PMC4711157 26386242

[81] Miranda LA Fischer RG Sztajnbok FR Johansson A Figueredo CM Gustafsson A . Increased interleukin-18 in patients with juvenile idiopathic arthritis and early attachment loss. J Periodontol. (2005) 76:75–82. doi: 10.1902/jop.2005.76.1.75 15830640

[82] Sete MR Figueredo CM Sztajnbok F . Periodontitis and systemic lupus erythematosus. Rev Bras Reumatol Engl Ed. (2016) 56:165–70. doi: 10.1016/j.rbre.2015.09.001 27267530

[83] Jing L Kim S Sun L Wang L Mildner E Divaris K . IL-37- and IL-35/IL-37-producing plasma cells in chronic periodontitis. J Dent Res. (2019) 98:813–21. doi: 10.1177/0022034519847443 PMC6589897 31050915

[84] Bae SC Lee YH . Causal association between periodontitis and risk of rheumatoid arthritis and systemic lupus erythematosus: a Mendelian randomization. Z Rheumatol. (2020) 79:929–36. doi: 10.1007/s00393-019-00742-w 31965238

[85] Hammoudeh M Al-Momani A Sarakbi H Chandra P Hammoudeh S . Oral manifestations of systemic lupus erythematosus patients in Qatar: A pilot study. Int J Rheumatol. (2018) 2018:6052326. doi: 10.1155/2018/6052326 29849650 PMC5914093

[86] Ugarte A Danza A Ruiz-Irastorza G . Glucocorticoids and antimalarials in systemic lupus erythematosus: an update and future directions. Curr Opin Rheumatol. (2018) 30:482–9. doi: 10.1097/BOR.0000000000000527 29870497

[87] Rezvaninejad R Dadmehr M Rezvaninejad R . Prevalence of oral manifestations in systemic lupus erythematosus patients referred to shahid mohammadi hospital in 2018 - 2019. Jundishapur J Health Sci. (2021) 13:e116144. doi: 10.5812/jjhs

[88] MaysJW SarmadiM MoutsopoulosNM . Oral manifestations of systemic autoimmune and inflammatory diseases: diagnosis and clinical management. J Evid Based Dent Pract. (2012) 12:265–82. doi: 10.1016/S1532-3382(12)70051-9 23040353

[89] Afilal S Rkain H Allaoui A Fellous S Berkchi JM Taik FZ . Oral hygiene status in rheumatoid arthritis patients and related factors. Mediterr J Rheumatol. (2021) 32:249–55. doi: 10.31138/mjr.32.3.249 PMC8693299 34964029

[90] Nazir MA Izhar F Akhtar K Almas K . Dentists' awareness about the link between oral and systemic health. J Family Community Med. (2019) 26:206–12. doi: 10.4103/jfcm.JFCM_55_19 PMC6755758 31572052

[91] Sales L Vassalo S Chaves M.D.G.A.M. Aarestrup FM . Periodontal disease and systemic lupus erythematosus activity. Rev Interdiscip Estud Exp. (2009) 1:14–20.

[92] Gofur NRP Nurdiana N Kalim H Handono K . Periodontitis is associated with disease severity and anti-double stranded DNA antibody and interferon-gamma levels in patients with systemic lupus erythematosus. J Taibah Univ Med Sci. (2019) 14:560–5. doi: 10.1016/j.jtumed.2019.09.005 PMC6940663 31908645

[93] Fabbri C Fuller R Bonfá E Guedes LK D'alleva PS Borba EF . Periodontitis treatment improves systemic lupus erythematosus response to immunosuppressive therapy. Clin Rheumatol. (2014) 33:505–9. doi: 10.1007/s10067-013-2473-2 24415114

[94] Ramos PS Criswell LA Moser KL Comeau ME Williams AH Pajewski NM . A comprehensive analysis of shared loci between systemic lupus erythematosus (SLE) and sixteen autoimmune diseases reveals limited genetic overlap. PloS Genet. (2011) 7:e1002406. doi: 10.1371/journal.pgen.1002406 22174698 PMC3234215

[95] Karydis A Sandal I Luo J Prislovsky A Gamboa A Rosloniec EF . Influence of the shared epitope on the elicitation of experimental autoimmune arthritis biomarkers. PloS One. (2021) 16:e0250177. doi: 10.1371/journal.pone.0250177 33857232 PMC8049293

[96] Barker TH Migliavaca CB Stein C Colpani V Falavigna M Aromataris E . Conducting proportional meta-analysis in different types of systematic reviews: a guide for synthesisers of evidence. BMC Med Res Method. (2021) 21:189. doi: 10.1186/s12874-021-01381-z PMC8451728 34544368

[97] Eke PI Page RC Wei L Thornton-Evans G Genco RJ . Update of the case definitions for population-based surveillance of periodontitis. J Periodontol. (2012) 83:1449–54. doi: 10.1902/jop.2012.110664 PMC6005373 22420873

[98] Ajeganova S Huizinga TW . Rheumatoid arthritis: Seronegative and seropositive RA: alike but different? Nat Rev Rheumatol. (2015) 11:8–9. doi: 10.1038/nrrheum.2014.194 25403158

[99] Anders HJ Saxena R Zhao MH Parodis I Salmon JE Mohan C . Lupus nephritis. Nat Rev Dis Primers. (2020) 6:7. doi: 10.1038/s41572-019-0141-9 31974366

[100] Hill AB . The environment and disease: association or causation? Proc R Soc Med. (1965) 58:295–300. doi: 10.1177/003591576505800503 14283879 PMC1898525

