# Supplementary material for: Higher odds of periodontitis in systemic lupus erythematosus compared to controls and rheumatoid arthritis: a systematic review, meta-analysis and network meta-analysis
Source: Front Immunol. 2024 Apr 2;15:1356714. doi: 10.3389/fimmu.2024.1356714 (PMC11019014; doi:10.3389/fimmu.2024.1356714)

**SUPPLEMENTARY DATA**

**Higher Odds of Chronic Periodontitis in Systemic Lupus Erythematosus Compared to Controls and Rheumatoid Arthritis: A Systematic Review, Meta-Analysis and Network Meta-Analysis**

Ping Ren Tan, Aaron J. L. Lee, Joseph J. Zhao, Yiong Huak Chan, Jia Hui Fu, Margaret Ma and Sen Hee Tay

**Supplemental Table 1.** Search strategies in used each database.

| **Database** | **Search terms for RA and periodontitis** | **Search terms for SLE and periodontitis** |
| --- | --- | --- |
| PubMed | (("rheumatoid arthritis") OR (arthritis, rheumatoid[MeSH Terms])) AND (("Periodontitis") OR (Periodontitis[MeSH Terms]) OR ("Pericementitis") OR ("chronic periodontitis"[MeSH Terms]) OR ("chronic periodontitis") OR ("periodontitis, chronic") OR ("adult periodontitis") OR ("periodontitis, adult")) | ((lupus erythematosus, systemic[MeSH Terms]))  AND (("Periodontitis") OR (Periodontitis[MeSH Terms]) OR ("Pericementitis") OR ("chronic periodontitis"[MeSH Terms]) OR ("chronic periodontitis") OR ("periodontitis, chronic") OR ("adult periodontitis") OR ("periodontitis, adult")) |
| Embase | (("rheumatoid arthritis") OR (arthritis, rheumatoid)) AND (("Periodontitis") OR (Periodontitis) OR ("Pericementitis") OR ("chronic periodontitis") OR ("chronic periodontitis") OR ("periodontitis, chronic") OR ("adult periodontitis") OR ("periodontitis, adult")) | ((lupus erythematosus, systemic))  AND (("Periodontitis") OR (Periodontitis) OR ("Pericementitis") OR ("chronic periodontitis") OR ("chronic periodontitis") OR ("periodontitis, chronic") OR ("adult periodontitis") OR ("periodontitis, adult")) |
| Scopus | (("rheumatoid arthritis") OR (arthritis, rheumatoid)) AND (("Periodontitis") OR (Periodontitis) OR ("Pericementitis") OR ("chronic periodontitis") OR ("chronic periodontitis") OR ("periodontitis, chronic") OR ("adult periodontitis") OR ("periodontitis, adult")) | ((lupus erythematosus, systemic))  AND (("Periodontitis") OR (Periodontitis) OR ("Pericementitis") OR ("chronic periodontitis") OR ("chronic periodontitis") OR ("periodontitis, chronic") OR ("adult periodontitis") OR ("periodontitis, adult")) |

**Supplemental Table 2.** Characteristics of the RA studies included in the meta-analysis. Data are mean +/- SD or frequency (%), unless otherwise specified.

| **Study (1^st^ Author, Year, Reference)** | **Country/Study Design** | **Sample Size, n** | **Type of Control** | **RA Classification Criteria** | **Periodontitis Definition** | **Immunosuppressants** | **Age** | **Female Gender, n (%)** | **Severity of CP** | **Quality Assessment** |
| --- | --- | --- | --- | --- | --- | --- | --- | --- | --- | --- |
| Ding, 2022 | China/Cross-Sectional | RA: 40  Control: 0 | No control | ACR/EULAR 2010 | American Academy of Periodontology’s classification criteria 2018 | N.A. | Categorised | RA: 21 (53) | N.A. | 8 |
| Xiao, 2021 | China/Cross-Sectional | RA: 307  Control: 324 | Healthy | 1987 ACR | PDI score of 4–6 and a positive BOP result | NSAIDs or immunosuppressant | Controls: 41.93 (+/-11.52)  RA: 40.36 (+/-10.13) | RA: 179 (58)  Controls: 173 (63) | N.A. | 8 |
| Inanc, 2021 [37] | Turkey/Case Control | RA: 55  Control: 58 | Healthy | 1987 ACR | ≥2 interproximal sites with CAL values ≥ 4 mm (not on the same tooth) | Non-methorexate or methorexate. | Controls: 40.8 (+/-11.98)  RA: 48.84 (+/-11.42) | RA: 41 (75)  Controls: 40 (69) | N.A. | 7 |
| Rodriguez, 2021 [47] | Colombia/Cross-Sectional | RA: 51  Control: 51 | Healthy | ACR/EULAR 2010 | 2017 World Workshop on the Classification of Periodontal and Peri-Implant Diseases and Conditions | N.A. | Controls: 48.55 (+/-10.92)  RA: 48.55 (+/-11.07) | RA: 41 (80)  Controls: 41(80) | RA stage I : 6, stage II :21, stage III : 10 stage IV : 14 Controls stage I : 5, stage II :27, stage III : 4 stage IV : 15. | 7 |
| Tar, 2021 [36] | Hungary/Case Control | RA: 23  Control: 17 | Healthy | ACR/EULAR 2010 | Based on previous studies and according to classification criteria published by Lang and Bartold and Caton et al. | Corticosteroid, Methorexate, biologics( no JAK inhibitors) biologics | Controls: 55.6 (+/-6.9)  RA: 57.3 (+/-11.0) | RA: 21 (91)  Controls: 16 (94) | N.A. | 6 |
| Cheah, 2020 [41] | Malaysia/Cross-Sectional | RA: 49  Control: 55 | Non-RA | ACR/EULAR 2010 | CDC/AAP | N.A. | Categorised | RA: 40 (82)  Controls: 34 (62) | N.A. | 7 |
| Han, 2020 [56] | Malaysia/Cross-Sectional | RA: 87  Control: 100 | Healthy | ACR/EULAR 2010 | CDC/AAP | N.A. | Categorised | RA: 74 (85)  Controls: 60 (60) | N.A. | 8 |
| Lee, 2020 [51] | Korea/Case Control | RA: 29840  Control: 529440 | Non-RA | N.A. | ICD code K05.3; Chronic periodontitis | N.A. | Categorised | RA: 21440 (72)  Controls: 260988 (49) | N.A. | 6 |
| Nguyen, 2020 [49] | Vietnam/Cross-Sectional | RA: 150  Control: 150 | OA | ACR/EULAR 2010 | CDC/AAP | Corticosteroid | Controls: 52.9 (+/-11.5)  RA: 51.7 (+/-10.4) | RA: 123 (82)  Controls: 126 (84) | N.A. | 8 |

Abbreviations: RA, rheumatoid arthritis; 1987 ACR, 1987 American College of Rheumatology classification criteria for RA; ACR/EULAR 2010, American College of Rheumatology/European League Against Rheumatism classification criteria for RA; CP, chronic periodontitis; OA, osteoarthritis; DMARDs, disease modifying anti rheumatic drugs; NSAIDs, non steroidal anti inflammatory drugs; N.A., not available; PDI, periodontal disease index; PI, periodontal index; GI, gingival index; CAL, clinical attachment loss; BOP, bleeding on probing; PD, probing depth; PPD, pocket probing depth; CDC/AAP, Centers for Disease Control and Prevention in partnership with the American Academy of Periodontology.

**Supplemental Table 2.** Characteristics of included RA studies included in the meta-analysis (continued).

| **Study (1^st^ Author, Year, Reference)** | **Country/Study Design** | **Sample Size, n** | **Type of Control** | **RA Classification Criteria** | **Periodontitis Definition** | **Immunosuppressants** | **Age** | **Female Gender, n (%)** | **Severity of CP** | **Quality Assessment** |
| --- | --- | --- | --- | --- | --- | --- | --- | --- | --- | --- |
| Renvert, 2020 [39] | Sweden/Cross-Sectional | RA: 126  Control: 249 | Non-RA | 1987 ACR, ACR/EULAR 2010 | Clinical presence of BOP at > 20% of recorded tooth surfaces, presence of > 2 non-adjacent sites with a PPD ≥ 5 mm, presence of bone loss at ≥2 sites with a distance between cement enamel junction-to bone level of ≥5 mm, or if evidence of a furcation invasion at molar teeth was found either clinically (grade II), or clearly visible on panoramic radiographs. | Reported : Corticosteroid, Methotrexate, DMARDs, biologics, NSAID | Controls: 70 (+/-7.1)  RA: 70 (+/-6.6) | RA: 88 (70)  Controls: 139 (56) | N.A. | 7 |
| Schulz, 2020 [45] | Germany/Case Control | RA: 111  Control: 256 | Healthy | ACR/EULAR 2010 | A “level 1 periodontitis” was defined as presence of proximal attachment loss of ≥3 mm in ≥2 non-adjacent teeth.  A “level 2 periodontitis” was described as occurrence of proximal attachment loss of ≥5 mm in ≥30% of teeth present. | N.A. | Categorised | RA: 79 (71)  Controls: 147 (57) | N.A. | 7 |
| Shrestha, 2020 [44] | Nepal/Cross-Sectional | RA: 43  Control: 0 | No control | ACR/EULAR 2010 | Site-specific thresholds for increased PD that have ranged from 3mm to ≥ 6 mm, and those for CAL have ranged from 2 mm to ≥ 6 mm. | N.A. | RA: 50.41 (+/-9.48) | RA: 32 (74) | RA mild : 4, moderate : 24, severe : 9 | 5 |
| Alhabashneh, 2019 [40] | Jordan/Case Control | RA: 102  Control: 100 | Non-RA | 1987 ACR | ≥ 4 teeth with ≥ 1 site with PPD ≥ 4mm and CAL ≥3mm | N.A. | Categorised | RA: 84 (82)  Controls: 80 (80) | N.A. | 7 |
| Corrêa, 2019 [61] | Brazil/Case Control | RA: 42  Control: 47 | Non-RA | ACR/EULAR 2010 | ≥2 interproximal sites with PD ≥ 4 mm or one site with PD ≥ 5 mm | N.A. | Categorised | RA: 18 (43)  Controls: 19 (40) | N.A. | 8 |
| deAzevedoBranco, 2019 [60] | Brazil/Cross-Sectional | RA: 42  Control: 70 | Non-RA | ACR/EULAR 2010 | ≥2 interproximal sites with PD ≥ 4 mm or one site with PD ≥ 5 mm | N.A. | Controls: 41.34 (+/-13.87)  RA: 52.04 (+/-11.08) | RA: 37 (88)  Controls: 54 (77) | N.A. | 8 |
| Kim, 2019 [54] | Korea/Cross-Sectional | RA: 157  Control: 20140 | Non-RA | N.A. | Periodontal PD ≥ 4 mm in at least one of the six sections | N.A. | Controls: 43.9 (+/-0.2)  RA: 56.3 (+/-1.4) | RA: 122 (78)  Controls: 11576 (57) | N.A. | 7 |
| Rodriguez-Lozano, 2019 [46] | Spain/Case Control | RA: 187  Control: 157 | OA | ACR/EULAR 2010 | Level 1, presence of proximal attachment and loss ≥ 3 mm in ≥ 2 nonadjacent teeth; and Level 2, presence of proximal attachment loss ≥ 5 mm in ≥ 30% of teeth | Reported:  Corticosteroid, DMARDs | Controls: 55.5 (+/-23.7)  RA: 54.4 (+/-10.8) | RA: 147 (79)  Controls: 101 (64) | RA level 0 : 5, level 1 : 98, level 2 : 84 Controls level 0 : 53, level 1 : 85, level 2 : 19 | 6 |
| Zhao, 2019 [42] | China/Case Control | RA: 128  Control: 109 | Healthy | ACR/EULAR 2010 | Mild periodontitis ≤30% bone loss and minimal or no BOP; moderate periodontitis ≤50% bone loss, BOP, and tooth mobility <2; severe periodontitis ≥50% bone loss, marked BOP, and tooth mobility ≥2 | Reported :  Corticosteroid, DMARDs, NSAIDs | N.A. | N.A. | N.A. | 7 |

Abbreviations: RA, rheumatoid arthritis; 1987 ACR, 1987 American College of Rheumatology classification criteria for RA; ACR/EULAR 2010, American College of Rheumatology/European League Against Rheumatism classification criteria for RA; CP, chronic periodontitis; OA, osteoarthritis; DMARDs, disease modifying anti rheumatic drugs; NSAIDs, non steroidal anti inflammatory drug; N.A., not available; PDI, periodontal disease index; PI, periodontal index; GI, gingival index; CAL, clinical attachment loss; BOP, bleeding on probing; PD, probing depth; PPD, pocket probing depth; CDC/AAP, Centers for Disease Control and Prevention in partnership with the American Academy of Periodontology.

**Supplemental Table 2.** Characteristics of included RA studies included in the meta-analysis (continued).

| **Study (1^st^ Author, Year, Reference)** | **Country/Study Design** | **Sample Size, n** | **Type of Control** | **RA Classification Criteria** | **Periodontitis Definition** | **Immunosuppressants** | **Age** | **Female Gender, n (%)** | **Severity of CP** | **Quality Assessment** |
| --- | --- | --- | --- | --- | --- | --- | --- | --- | --- | --- |
| Mikuls, 2018 [38] | United States/Case Control | RA: 260  Control: 296 | OA | 1987 ACR | CAL greater than or equal to 6 mm in 2 or more teeth and one or more sites with PPD greater than or equal to 5 mm | Reported : Corticosteroid, Methotrexate, biologic | Controls: 60 (+/-11)  RA: 59 (+/-12) | RA: 195 (75)  Controls: 235 (79) | N.A. | 5 |
| Ayravainen, 2017 [64] | Finland/Case Control | RA: 81  Control: 43 | Non-RA | N.A. | CDC/AAP | Reported : Corticosteroid, DMARD | Controls: 56 (+/-13)  RA: categorised | RA: 68 (84)  Controls: 38 (88) | Reported : with baseline and follow-up changes for RA and controls. | 6 |
| Kirchner, 2017 [53] | Germany/Cross-Sectional | RA: 103  Control: 104 | Healthy | 1987 ACR | Page and Eke Journal of Periodontol 2007 | Reported : Corticosteroid, DMARDs, biologic | Controls: 56.73 (+/-11.88)  RA: 55.48 (+/-10.98) | RA: 58 (56)  Controls: 68 (65) | RA no/mild : 36, moderate : 54, severe : 13 Control no/mild : 25, moderate : 55, severe : 24 | 5 |
| Choi, 2016 [62] | Korea/Cross-Sectional | RA: 264  Control: 88 | Non-RA | 1987 ACR | American Academy of Periodontology classification | Reported : Corticosteroid, NSAID, biologic | Controls: 58.2 (+/-11.6)  RA: 58.2 (+/-12.0) | RA: 231 (88)  Controls: 77 (88) | RA mild : 96, moderate : 161, severe : 7 Control mild : 58, moderate : 30, severe : 0 | 7 |

Abbreviations: RA, rheumatoid arthritis; 1987 ACR, 1987 American College of Rheumatology classification criteria for RA; ACR/EULAR 2010, American College of Rheumatology/European League Against Rheumatism classification criteria for RA; CP, chronic periodontitis; OA, osteoarthritis; DMARDs, disease modifying anti rheumatic drugs; NSAIDs, non steroidal anti inflammatory drug; N.A., not available; PDI, periodontal disease index; PI, periodontal index; GI, gingival index; CAL, clinical attachment loss; BOP, bleeding on probing; PD, probing depth; PPD, pocket probing depth; CDC/AAP, Centers for Disease Control and Prevention in partnership with the American Academy of Periodontology.

**Supplemental Table 2.** Characteristics of included RA studies included in the meta-analysis (continued).

| **Study (1^st^ Author, Year, Reference)** | **Country/Study Design** | **Sample Size, n** | **Type of Control** | **RA Classification Criteria** | **CP Diagnosis Criteria** | **Immunosuppressants** | **Age** | **Female Gender, n (%)** | **Severity of CP** | **Quality Assessment** |
| --- | --- | --- | --- | --- | --- | --- | --- | --- | --- | --- |
| Eriksson, 2016 [58] | Sweden/Case Control | RA: 2343  Control: 3386 | Non-RA | 1987 ACR | Diagnostic code from the Swedish National Dental Health Registry | N.A. | Categorised | RA: 1719 (73)  Controls: 2469 (73) | N.A. | 7 |
| Laugisch, 2016 [52] | Switzerland/Case Control | RA: 52  Control: 44 | Non-RA | ACR/EULAR 2010 | PD ≥3.5 mm, plaque or calculus accumulation, and BOP in at least two sextants | Reported : Corticosteroid, DMARD | Controls: 53 (+/-12)  RA: 57 (+/-11) | RA: 41 (79)  Controls: 26 (59) | N.A. | 5 |
| Gabarrini, 2015 [57] | Netherlands/Case Control | RA: 12  Control: 80 | Non-RA | N.A. |  | Reported : Methotrexate | Median, IQR  Controls: 51 (42-60)  RA: 64 (56-71) | RA: 9 (75)  Controls: 43 (54) | N.A. | 6 |
| Lee, 2015 [35] | Korea/Cross-Sectional | RA: 248  Control: 85 | Healthy | 1987 ACR | American Academy of Periodontology 2004 classification | N.A. | Controls: 59.13 (+/-1.3)  RA: 60.1 (+/-0.7) | RA: 218 (88)  Controls: 74 (87) | RA mild : 88, moderate : 153, severe : 7 Control mild : 57, moderate : 28, severe : 0 | 8 |
| Mikuls, 2014 [50] | United States/Case Control | RA: 287  Control: 330 | OA | 1987 ACR | CAL of ≥ 6 mm on ≥ 2 teeth, and one or more sites with PD of ≥ 5 mm | Reported : Corticosteroid, DMARDs, biologic | Controls: 59 (+/-11)  RA: 59 (+/-12) | RA: 106 (37)  Controls: 132 (40) | N.A. | 5 |
| Chen, 2013 [63] | Taiwan/Case Control | RA: 13779  Control: 137790 | Non-RA | 1987 ACR | Diagnostic code from National Health Insurance Programme | N.A. | Categorised | RA: 10659 (77)  Controls: 106590 (77) | N.A. | 9 |
| Joseph, 2013 [55] | India/Case Control | RA: 100  Control: 112 | Non-RA | 1987 ACR | International Workshop for a Classification of Periodontal Diseases and Conditions, 1999 clinical attachment score | Reported : DMARDs, NSAIDs | Controls: 45.91 (+/-9.76)  RA: 46.54 (+/-8.5) | RA: 76 (76)  Controls: 96 (86) | RA mild : 42, moderate : 48, severe : 10 Control mild : 84, moderate : 6, severe : 2 | 5 |

Abbreviations: RA, rheumatoid arthritis; 1987 ACR, 1987 American College of Rheumatology classification criteria for RA; ACR/EULAR 2010, American College of Rheumatology/European League Against Rheumatism classification criteria for RA; CP, chronic periodontitis; OA, osteoarthritis; DMARDs, disease modifying anti rheumatic drugs; NSAIDs, non steroidal anti inflammatory drug; N.A., not available; PDI, periodontal disease index; PI, periodontal index; GI, gingival index; CAL, clinical attachment loss; BOP, bleeding on probing; PD, probing depth; PPD, pocket probing depth; CDC/AAP, Centers for Disease Control and Prevention in partnership with the American Academy of Periodontology.

**Supplemental Table 2.** Characteristics of included RA studies included in the meta-analysis (continued).

| **Study (1^st^ Author, Year, Reference)** | **Country/Study Design** | **Sample Size, n** | **Type of Control** | **RA Classification Criteria** | **Periodontitis Definition** | **Immunosuppressants** | **Age** | **Female Gender, n (%)** | **Severity of CP** | **Quality Assessment** |
| --- | --- | --- | --- | --- | --- | --- | --- | --- | --- | --- |
| Susanto 2013 [43] | Indonesia/Case Control | RA: 75  Control: 75 | Non-RA | 1987 ACR | Page and Eke case definitions | Reported : Corticosteroid, DMARDs, NSAIDs | Controls: 46.9 (+/-11.2)  RA: 46.5 (+/-11.3) | RA: 60 (80)  Controls: 60 (80) | RA no/mild : 22, moderate : 37, severe : 16 Control no/mild : 23, moderate : 40, severe : 12 | 7 |
| deSmit, 2012 [59] | Netherlands/Case Control | RA: 95  Control: 420 | Non-RA | 1987 ACR | Dutch periodontal screening index | Reported : DMARDS, TNF antagonist | RA: 56 (+/-11) | RA: 65 (68) Controls: 285 (68) | RA no : 30, moderate : 43, severe : 27 Control no : 70, moderate : 18, severe : 12 | 8 |
| Okada, 2011 [48] | Japan/Case Control | RA: 80  Control: 38 | Non-RA | 1987 ACR | ≥ 1 periodontitis site with PD >3 mm and CAL ≥ 3 mm | Reported : Corticosteroid, DMARDs, NSAIDs, TNF antagonist | Controls: 57.4 (+/-1.4)  RA: 59.5 (+/-1.5) | RA: 70 (88)  Controls: 33 (87) | N.A. | 7 |
| Dissick, 2010 [19] | United States/Case Control | RA: 69  Control: 35 | OA | 1987 ACR | American Academy of Periodontology 2004 classification | Reported : Corticosteroid, DMARDs, biologic | Controls: 58 (+/-15)  RA: 62 (+/-12) | RA: 12 (17)  Controls: 5 (14) | N.A. | 6 |

Abbreviations: RA, rheumatoid arthritis; 1987 ACR, 1987 American College of Rheumatology classification criteria for RA; ACR/EULAR 2010, American College of Rheumatology/European League Against Rheumatism classification criteria for RA; CP, chronic periodontitis; OA, osteoarthritis; DMARDs, disease modifying anti rheumatic drugs; NSAIDs, non steroidal anti inflammatory drug; N.A., not available; PDI, periodontal disease index; PI, periodontal index; GI, gingival index; CAL, clinical attachment loss; BOP, bleeding on probing; PD, probing depth; PPD, pocket probing depth; CDC/AAP, Centers for Disease Control and Prevention in partnership with the American Academy of Periodontology.

**Supplemental Table 3.** Parameters for ascertainment of periodontitis**.**

| Periodontal index | Assessing the inflammation in the investing tissue or supporting tissue of the gingivae by the depth the probe is able to enter. |
| --- | --- |
| Gingival index | Severity of the inflammation of the gingivae based on the appearance of the gingivae such as reddish, edema, shiny inflammation and presence of blood. |
| Clinical attachment loss | Clinical attachment loss is the distance between the cementoenamel junction and the periodontal pocket base. |
| Bleeding on probing | Using a dental probe on the 4 sides of the tooth surface, mesial, distal, buccal and palatal/lingual to assess for bleeding. If bleeding is found a positive sign |
| Plaque index | The examination was using six teeth, namely 16, 12, 24, 36, 32 and 44 to assess for plaque in the gingival area. |
| Calculus index | To look for calculus and extrinsic staining on specific teeth. |
| Mobility of tooth | Examination of mobility teeth using two mouth glasses, any movement more than 0 degrees is positive. |

**Supplemental Table 4.**  Studies reporting disease activity in SLE patients and prevalence/severity of periodontitis.

| **Study (Author, Year)** | **Disease activity measure** | **Disease activity** | **Prevalence of periodontitis** | **Severity of periodontitis** |
| --- | --- | --- | --- | --- |
| Mendonca *et al.*, 2019 | SLEDAI-2K | 4.00 (0.00-18.00) | 66% | Not reported |
| Calderaro *et al.*, 2017 | SLEDAI-2K | 4.00 (2.00-7.00) | 68% | Mild : 2, moderate : 36, severe : 13 |
| Correa *et al.*, 2017 | SLEDAI-2K | 4.50 ± 4.00 | 67.3% | Not reported |
| Zhang *et al.*, 2017 | SLEDAI-2K | 9.07 ± 5.75 | 83.3% | Mild : 18, moderate : 46, severe : 26 |

Abbreviations: SLE, systemic lupus erythematosus; SLEDAI-2K, Systemic Lupus Erythematosus Disease Activity Index 2000.

**Supplemental Table 5.** Studies reporting disease activity in RA patients and prevalence/severity of periodontitis.

| **Study** | **DAS type** | **DAS score**  **(mean, SD)** | **Prevalence of periodontitis** | **Severity of periodontitis** |
| --- | --- | --- | --- | --- |
| deSmit *et al.*, 2012 | DAS28-ESR | 2.40 ± 0.93 | 70% | No : 30, moderate : 43, severe : 27 |
| Choi *et al.*, 2016 | DAS28-ESR | 3.30 ± 1.40 | 100% | Mild : 96, moderate : 161, severe : 7 |
| Correa *et al.*, 2019 | DAS28-ESR | 3.70 ± 1.50 | 50% | Not reported |
| Rodriguez-Lozano *et al.*, 2019 | DAS28-ESR/ DAS28-CRP | 3.81 ± 1.31/3.18 ± 1.18 | 97.33% | Level 0 : 5, level 1 : 98, level 2 : 84 |
| Mikuls *et al.*, 2014 | DAS28-CRP | 3.20 ± 1.30 | 34.8% | Not reported |
| Mikuls *et al.*, 2018 | DAS28-CRP | 3.30 ± 1.30 | 38% | Not reported |

Abbreviations: DAS, disease activity score; ESR, erythrocyte sedimentation rate; DAS28-ESR, disease activity score-28 for rheumatoid arthritis with erythrocyte sedimentation rate; DAS28-CRP, disease activity score-28 for rheumatoid arthritis with C-reactive protein; RA, rheumatoid arthritis.

**Supplemental Figure 1.** Pooled prevalences of periodontitis in controls of RA and SLE studies.


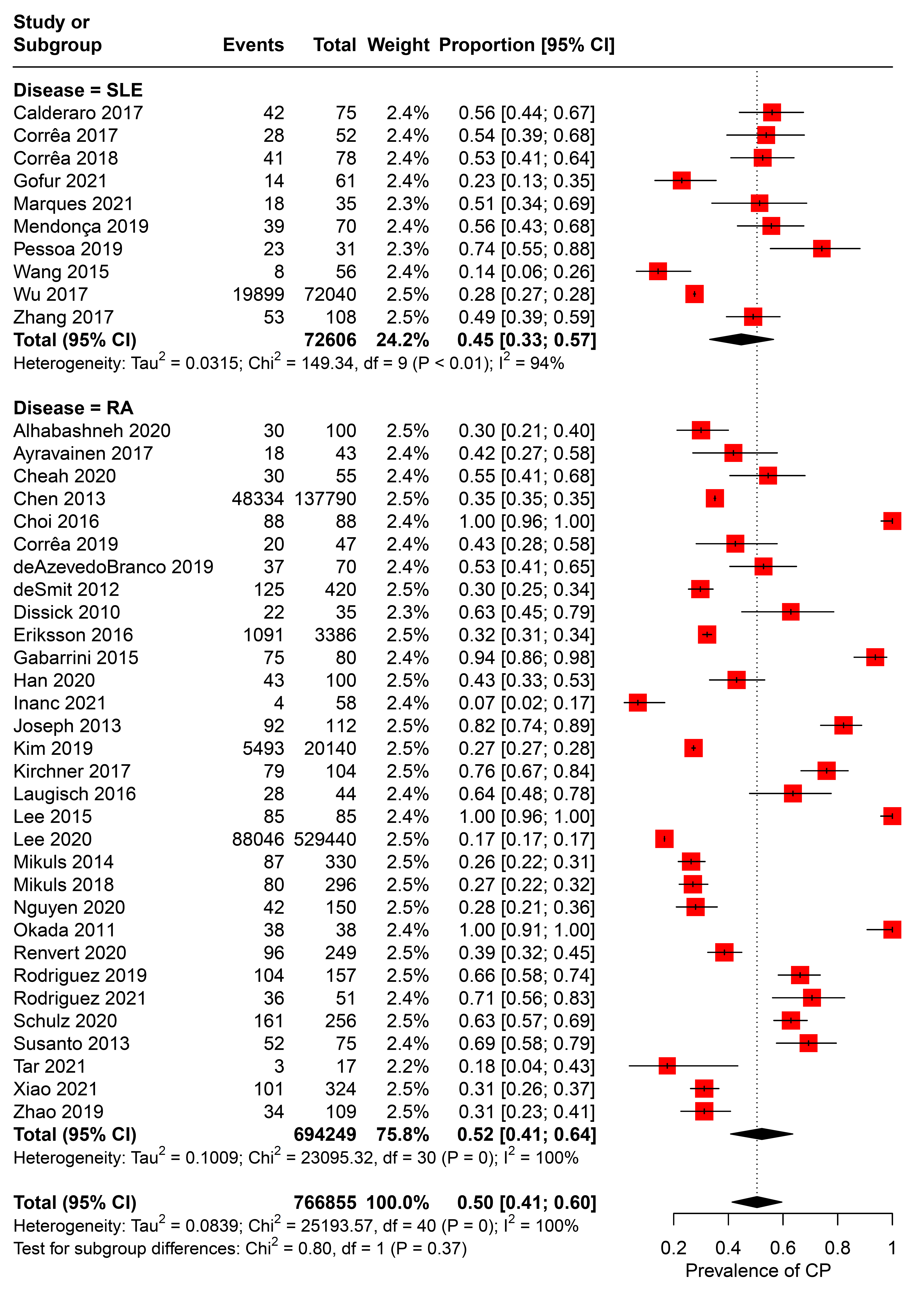

Supplement: Supplementary file 1 [file DataSheet_1.docx]
